# Posttranslational modifications of proteins are key features in the identification of CSF biomarkers of multiple sclerosis

**DOI:** 10.1186/s12974-022-02404-2

**Published:** 2022-02-08

**Authors:** Ivan L. Salazar, Ana S. T. Lourenço, Bruno Manadas, Inês Baldeiras, Cláudia Ferreira, Anabela Claro Teixeira, Vera M. Mendes, Ana Margarida Novo, Rita Machado, Sónia Batista, Maria do Carmo Macário, Mário Grãos, Lívia Sousa, Maria João Saraiva, Alberto A. C. C. Pais, Carlos B. Duarte

**Affiliations:** 1 CNC-Center for Neuroscience and Cell Biology, University of Coimbra, Coimbra, Portugal; 2 Institute for Interdisciplinary Research, University of Coimbra, Coimbra, Portugal; 3 Faculty of Medicine, University of Coimbra, Coimbra, Portugal; 4 Coimbra Chemistry Centre, Department of Chemistry, University of Coimbra, Coimbra, Portugal; 5 Molecular Neurobiology Group, Instituto de Biologia Molecular e Celular (IBMC), Instituto de Investigação e Inovação em Saúde (i3S), University of Porto, Porto, Portugal; 6 Neurology Department, CHUC-Centro Hospitalar e Universitário de Coimbra, Coimbra, Portugal; 7 Biocant-Associação de Transferência de Tecnologia, Cantanhede, Portugal; 8 Department of Life Sciences, University of Coimbra, Coimbra, Portugal

**Keywords:** Multiple sclerosis, Biomarkers, Cerebrospinal fluid (CSF), Transthyretin (TTR)

## Abstract

**Background:**

Multiple sclerosis is an inflammatory and degenerative disease of the central nervous system (CNS) characterized by demyelination and concomitant axonal loss. The lack of a single specific test, and the similarity to other inflammatory diseases of the central nervous system, makes it difficult to have a clear diagnosis of multiple sclerosis. Therefore, laboratory tests that allows a clear and definite diagnosis, as well as to predict the different clinical courses of the disease are of utmost importance. Herein, we compared the cerebrospinal fluid (CSF) proteome of patients with multiple sclerosis (in the relapse–remitting phase of the disease) and other diseases of the CNS (inflammatory and non-inflammatory) aiming at identifying reliable biomarkers of multiple sclerosis.

**Methods:**

CSF samples from the discovery group were resolved by 2D-gel electrophoresis followed by identification of the protein spots by mass spectrometry. The results were analyzed using univariate (Student’s *t* test) and multivariate (Hierarchical Cluster Analysis, Principal Component Analysis, Linear Discriminant Analysis) statistical and numerical techniques, to identify a set of protein spots that were differentially expressed in CSF samples from patients with multiple sclerosis when compared with other two groups. Validation of the results was performed in samples from a different set of patients using quantitative (e.g., ELISA) and semi-quantitative (e.g., Western Blot) experimental approaches.

**Results:**

Analysis of the 2D-gels showed 13 protein spots that were differentially expressed in the three groups of patients: Alpha-1-antichymotrypsin, Prostaglandin-H2-isomerase, Retinol binding protein 4, Transthyretin (TTR), Apolipoprotein E, Gelsolin, Angiotensinogen, Agrin, Serum albumin, Myosin-15, Apolipoprotein B-100 and EF-hand calcium-binding domain—containing protein. ELISA experiments allowed validating part of the results obtained in the proteomics analysis and showed that some of the alterations in the CSF proteome are also mirrored in serum samples from multiple sclerosis patients. CSF of multiple sclerosis patients was characterized by TTR oligomerization, thus highlighting the importance of analyzing posttranslational modifications of the proteome in the identification of novel biomarkers of the disease.

**Conclusions:**

The model built based on the results obtained upon analysis of the 2D-gels and in the validation phase attained an accuracy of about 80% in distinguishing multiple sclerosis patients and the other two groups.

**Supplementary Information:**

The online version contains supplementary material available at 10.1186/s12974-022-02404-2.

## Background

Multiple sclerosis (MS) is an inflammatory and degenerative disease of the central nervous system (CNS) affecting primarily young adults. The disease typically begins in early adulthood and has a female predominance of approximately 2:1 [1]. The clinical symptoms of MS are heterogeneous, including sensory disturbances, visual impairment, fatigue and reduced coordination, and its clinical course and prognosis are also variable [2]. The diagnosis of MS is difficult because of this heterogeneity and also because its signs and symptoms can be similar to many other medical problems [3–5]. These limitations in the clinical practice make studies aiming at identifying biological markers of MS extremely relevant.

Currently, there is no specific diagnostic test for MS. Although magnetic resonance imaging (MRI) scans of the brain and spinal cord, evoked potentials and cerebrospinal fluid (CSF) analysis can be of aid, the diagnosis is still based on clinical criteria [5, 6]. Along with epidemiological studies, neuroimaging has provided some insight into the natural course and prognosis factors of MS. However, the overall ability to predict different clinical courses of the disease, and its response to treatment, is still very limited. There is increasing evidence showing that the levels of neurofilament light chain in the CSF and serum can be used as indicators of prognosis and response to treatment in MS patients [7, 8]. However, there is still an emerging need to further characterize the disease by simple and reliable laboratory tests, not only to describe clinical disease activity at a given timepoint, but ideally also to be able to predict the future development, in response or not to treatment, of this disabling and partially asymptomatic disease [9].

The CSF is a highly valuable sample in the search for novel molecular biomarkers of neurodegenerative disorders. CSF represents a repertoire of neuro-secreted, biosynthesized and metabolized molecular products of the CNS. Diffusion of macromolecules from the peripheral circulatory system to the CSF is highly regulated by the blood–brain barrier, which prevents the uncontrolled distribution of proteins in the CNS [10]. Therefore, a comprehensive study of the CSF proteome represents an important step towards a better understanding of the disease and may lead to the identification of biomarkers, which can help diagnosing MS. Up to now, the routine study of the CSF for the diagnosis of MS has been almost exclusively limited to the characterization of the presence of oligoclonal bands [11]. Alterations in the CSF proteome detected in MS patients may be exploited by physicians as putative neuropathological-derived biomarkers.

Previous proteomics studies aiming at identifying alterations in the CSF proteome associated with MS gave highly diversified results, making it difficult to conclude about their biomarker potential. These limitations result, among other factors, from (i) the low number of patients analyzed, (ii) differences in sample handling and storage, (iii) the use of different control samples, (iv) the diversity of proteomics approaches used with distinct sensitivities and (v) the variety of methodologies used for data analysis [12–23]. Therefore, at this point, mandatory steps for standardization of preanalytical and analytical variables still remain to be identified for several biomarkers. Moreover, the combination of a single or a panel of biomarkers should be analyzed together with the clinical and imaging outcomes of each individual patient [24]. In addition, the intrinsic heterogeneity of MS makes sample size a critical issue in the design of this type of studies. Putative biomarkers for MS identified using proteomics approaches should also be validated using other experimental approaches, preferentially using experimental strategies that can be implemented in a clinical laboratory [25]. This validation step is missing in many studies (e.g., [13, 15, 17–22]), which makes difficult the assessment of the putative clinical relevance of the proposed biomarker proteins. Moreover, the applicability of discovered and validated biomarkers of MS in the clinical practice has been halted by the lack of multicentre validation of those molecules using large cohorts of patients [26].

When attempting to discover differences in protein expression and protein posttranslational modifications resulting from disease, the use of 2D electrophoresis coupled to mass spectrometry is a widely used and powerful analytical tool [27]. Therefore, in this work we resolved the CSF proteome from patients with relapse–remitting multiple sclerosis (RRMS; *n* = 69) and with other diseases of the CNS (other inflammatory and non-inflammatory) (*n* = 69) in two-dimensional gel electrophoresis, and the protein spots of interest were identified by mass spectrometry. The data set generated was analyzed using a combination of univariate (Student’s *t* test) and multivariate (Principal Component Analysis (PCA), Linear Discriminant Analysis (LDA)) statistical techniques, and allowed the identification of a set of proteins that are differentially expressed in CSF samples of patients with RRMS when compared with other inflammatory and non-inflammatory diseases of the CNS. The same experimental groups were used to validate the findings in the discovery groups, in a distinct cohort of patients. For this purpose, CSF and serum obtained from patients diagnosed with RRMS and other diseases of the CNS (other inflammatory and non-inflammatory) were subjected to ELISA analysis, and semi-denaturing gel electrophoresis, to determine their relative abundance and changes in protein migration, respectively. Once the proteins were selected and identified, the same machine learning approaches (Hierarchical Cluster Analysis [HCA], PCA and LDA) were performed, allowing for a better understanding of the individual impact of each protein in the discrimination of the different groups in the validation cohort.

## Materials and methods

### Clinical patient’s information

Patients diagnosed with RRMS, other inflammatory diseases and non-inflammatory diseases of the CNS were recruited at the Neurology Unit of the Coimbra University Hospital Center. RRMS patients and non-RRMS individuals were diagnosed according to the 2005 revision of the McDonald criteria [28], and subsequent revisions (when applicable, e.g., [6]). All samples used in this study were collected from patients followed in the the Neurology Unit of the Coimbra University Hospital Center, and had their initial diagnosis confirmed. The demographic and clinical features of the patients and for each body fluid (CSF and serum) are summarized in Tables 1 and 2. Patients with other inflammatory and non-inflammatory diseases were diagnosed by the clinicians based on the follow-up of the patients for the period required for definitive judgement, and according to each individual disease criteria (e.g., for Neuromyelitis optica spectrum disorders [29], Neuro-Behçet [30], Migraine [31] and Parkinson’s Disease [32]). This was a discovery/validation study using samples obtained from patients diagnosed with RRMS (193), and with other inflammatory (133), and non-inflammatory (174) diseases of the CNS. From these, we selected 69 patients with RRMS, 27 patients with other inflammatory diseases of the CNS, and 42 patients with non-inflammatory diseases of the CNS for the discovery cohort. All other patient samples were used as an independent validation cohort. This allowed to maintain complete independency in the two populations of patients used in the study.

**Table 1:** Demographic and clinical features of the patients included in the study: CSF samples

	Disease	Number of patients	Age (Mean ± SD)	Gender
Discovery	RRMS	69	36.9 ± 10.6	25M; 44F
	INF	27	46.4 ± 15.7	10M; 17F
	N-INF	42	45.3 ± 11.2	14M; 28F
Validation	RRMS	68	36.8 ± 9.9	18M; 50F
	INF	53	46.6 ± 15.6	20M; 33F
	N-INF	74	51.9 ± 11.9	29M; 45F

*M* male, *F* female, *RRMS* Relapse–remitting multiple sclerosis, *INF* other inflammatory diseases of the CNS, *N-INF* non-inflammatory diseases of the CNS

**Table 2:** Demographic and clinical features of the patients included in the study: Serum samples

	Disease	Number of patients	Age (Mean ± SD)	Gender
Validation	RRMS	56	35.5 ± 11.2	14M; 42F
	INF	53	46.7 ± 16.1	17M; 36F
	N-INF	58	51.3 ± 12.7	23M; 35F

*M* male, *F* female, *RRMS* Relapse–remitting multiple sclerosis, *INF* other inflammatory diseases of the CNS, *N-INF* non-inflammatory diseases of the CNS

**Fig. 1:**
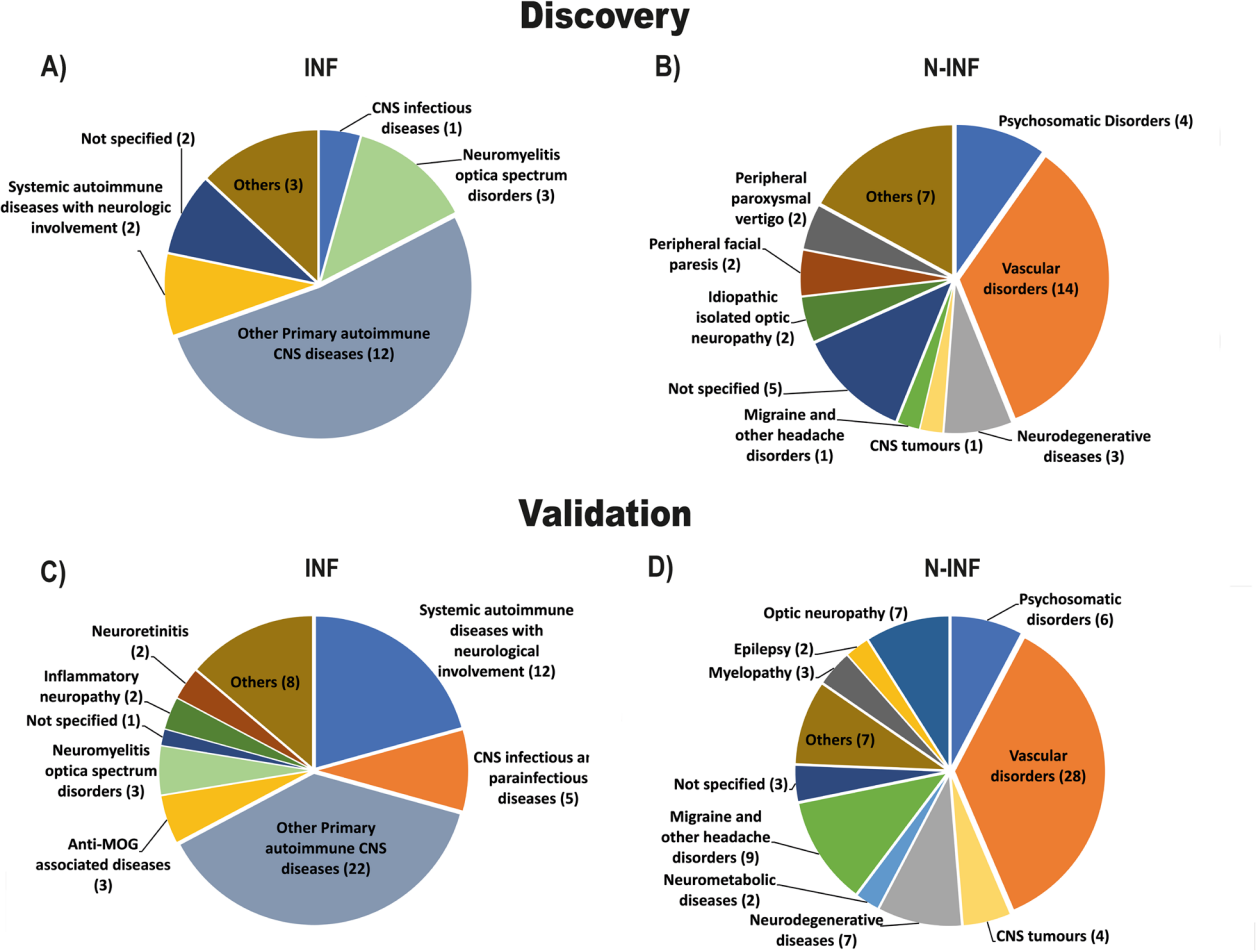
Pie chart summarizing the fraction of patients diagnosed with each individual inflammatory (INF) and non-inflammatory (N-INF) disease of the nervous system. **A** Diseases identified in patients from the discovery group diagnosed with an inflammatory disease of the CNS: CNS infectious diseases (Neurosyphilis), Other primary autoimmune diseases of CNS (CNS vasculitis, Encephalomyelitis, Idiopathic myelitis and Idiopathic optic neuritis), Systemic autoimmune diseases with neurologic involvement (Neuro-Behçet), and others (Harada’s disease). Two patients with Guillain–Barre and demyelinating inflammatory polyneuropathy were also included in the latter group. **B** Diseases identified in patients from the discovery group diagnosed with a non-inflammatory disease of the CNS: Psychosomatic Disorders (Fibromyalgia, Sensitive complains and Depression), Vascular disorders (Anterior ischemic optic neuropathy, White matter lesions caused by small vessel disease, Ischemic stroke and Transient ischemic attack), Neurodegenerative disorders (Parkinson’s disease, Alzheimer’s disease and Spastic paraparesis) and others (Cervical spondylotic myelopathy, D5 body hemangioma, Essential tremor, Normal pressure hydrocephalus, Ocular lesions and Rathke’s cleft cyst). One patient with Diabetic polyneuropathy was also included in the latter group. **C** Diseases identified in patients from the validation group  diagnosed with an inflammatory disease of the CNS: Systemic autoimmune diseases with neurologic involvement (Neurolupus, Neuro-Behçet, Neurosarcoidosis and Erdheim–Chester disease), CNS infectious and parainfectious diseases (Neurocysticercosis, Neuroborreliosis, Progressive Multifocal Leukoencephalopathy and Parainfectious Pancerebellum Syndrome), other primary autoimmune diseases of CNS (anti-GAD ataxia, CNS vasculitis, Encephalitis, Encephalomyelitis, Idiopathic monophasic myelitis, recurrent myelitis), anti-MOG associated disease (anti-MOG [myelin oligodendrocyte glycoprotein] disease and anti-MOG + optic neuritis), other diseases (Dysautonomic syndrome, Encephalopathy, Harada’s disease, Idiopathic inflammatory Myopathy, Pachymeningitis, PanUveitis, Rhombencephalitis and Spondylodiscitis). **D** Diseases identified in patients from the validation group diagnosed with a non-inflammatory disease of the CNS: Psychosomatic disorders (memory and sensitive complains), Vascular diseases (CADASIL, Cerebral thrombosis, Microvascular VI paresis, Ischemic stroke, Subdural hematoma and White matter lesions caused by small vessel disease), CNS tumors (Brainstem neoplasm, CNS metastasis and CNS B cells lymphoma), Neurodegenerative diseases (Progressive supranuclear palsy, Ataxia, Idiopathic chorea, Hereditary spastic paraplegia, Dementia and CANVAS), Neurometabolic diseases (Vanishing white matter disease and Marchiafava-Bignami disease) and other diseases (Dystonia, Encephalopathy, Hydrocephalus, Idiopathic leukoencephalopathy, Intracerebral hypertension, Reflex syncope and Stiff-Person syndrome)

The clinical diagnosis of the patients was performed as summarized in Fig. 1. In the discovery phase of the study, the characteristics of subjects included in the groups of patients with other inflammatory and non-inflammatory diseases of the CNS were the following: the former group of patients was comprised primarily by autoimmune diseases of the CNS (e.g., CNS vasculitis, Encephalomyelitis, Idiopathic myelitis, Idiopathic optic neuritis), whereas the latter group was dominated by patients diagnosed with vascular disorders (e.g., Anterior ischemic optic neuropathy, White matter lesions caused by small vessel disease, Ischemic stroke and Transient ischemic attack) and a mixture of other diseases (e.g., Cervical spondylitis myelopathy, D5 body haemangioma, Diabetic polyneuropathy, Essential tremor, Normal pressure hydrocephalus, Ocular lesions and Rathke’s cleft cyst). The samples used in the validation studies were obtained from patients with the following pathologies: (i) the group of patients with other inflammatory diseases of the CNS included mainly subjects with autoimmune diseases of the CNS (e.g., anti-GAD ataxia, CNS vasculitis, Encephalitis, Encephalomyelitis, Idiopathic monophasic myelitis and recurrent myelitis), Systemic autoimmune diseases with neurologic involvement (e.g., Neurolupus, Neuro-behçet, Neurosarcoidosis and Erdheim Chester disease), other pan-inflammatory diseases (e.g., Dysautonomic Syndrome, Encephalopathy, Harada’s disease, Idiopathic Inflammatory Myopathy, Pachymeningitis, PanUveitis, Rhombencephalitis, Spondylodiscitis) and CNS Infectious and parainfectious diseases (e.g., Neurocysticercosis, Neuroborreliosis, Progressive multifocal leucoencephalopathy, Parainfectious pancerebellum syndrome); (ii) patients with non-inflammatory diseases of the CNS included mainly subjects with vascular diseases (e.g., CADASIL, Cerebral thrombosis, Microvascular VI paresis, Ischemic stroke, Subdural hematoma, White matter lesions caused by small vessel disease), neurodegenerative diseases (e.g., Progressive supranuclear palsy, Ataxia, Idiopathic chorea, Hereditary spastic paraplegia, Dementia and CANVAS), and other pan-non-inflammatory diseases of the CNS (e.g., Dystonia, Encephalopathy, Hydrocephalus, Idiopathic leukoencephalopathy, Intracerebral hypertension, Reflex syncope and Stiff Woman Syndrome). For non-specified diseases, the definitive diagnosis was not accomplished. Nevertheless, the clinical information available allowed sorting these patients with inflammatory and non-inflammatory diseases of the CNS.

### Sample collection and preparation for electrophoresis

CSF and blood samples were collected from patients during their routine diagnostic evaluation, according to a standard operating procedure [33], before starting any immunomodulatory therapy. Briefly, CSF samples were collected in the morning into sterile polypropylene tubes (minimum volume of collection- 6 mL). Samples were centrifuged at 2000×*g*, for 10 min at 4 °C within 2 h of collection, and the supernatant was separated and aliquoted into 2 mL polypropylene cryotubes. CSF aliquots were stored frozen at − 80 °C within 4 h of collection and kept frozen until analysis. Paired blood samples were obtained at the same day as CSF samples. Blood was collected into serum separation tubes, allowed to stand for 30 min and then centrifuged at 2000×*g*, for 10 min at 4 °C. The obtained serum was then aliquoted into 2 mL polypropylene cryotubes, stored at − 80 °C within 4 h of collection, and kept frozen until analysis.

All the samples were subjected to ultrafiltration at 16,100×*g*, for 20 min at 4 °C, using 5 kDa cutoff filters (Vivaspin 5000 MWCO PES) to remove possible contaminants, such as salts, nucleic acids and lipids, which interfere with protein separation in 2D electrophoresis. Filtered samples were solubilized in a sample buffer composed by 6 M urea (USB), 1.5 M thiourea (Sigma-Aldrich), 3% (v/v) CHAPS (USB), 1.2% (v/v) DeStreak (GE Healthcare), 1.5% (v/v) IPG buffer (GE Healthcare) and bromophenol blue. After solubilization, CSF samples were kept on ice and sonicated to improve protein recovery [34], using a 3 mm stepped microtip with a Vibra Cell System (Sonics and materials), in five cycles of 10 s, each consisting of 5 s sonication followed by a 5 s interval (to keep the samples at low temperature). Each sonication step was performed with increasing amplitude, starting from zero, and the amplitude was maintained below 40 kHz. Protein concentration was determined by 2-D Quant Kit (GE Healthcare).

### 2D electrophoresis

One hundred and forty micrograms of protein were actively rehydrated for 12 h at 50 V using pH 4–7 IPG strips (GE Healthcare). IEF was performed as follows: 500 V (500 Vh step and hold (SH)), 1000 V (1000 Vh SH), 10,000 V (15,000 Vh with linear increase), and final focusing at 10,000 V during 14 h, using Protean IEF cell (Bio-Rad), with a current limited at 50 μA per strip. Prior to SDS-PAGE the IPG strips were equilibrated to SDS for 15 min in a reducing equilibration buffer (50 mM Tris–HCL pH 8.8, 30% (v/v) glycerol (Sigma), 2% SDS (Bio-Rad)) in the presence of 1% (m/v) dithiothreitol (USB Chemicals), followed by an additional step in an alkylation equilibrium solution containing 2.5% (m/v) iodoacetamide (Merck). The IPG strips were then placed on the top of a 10% polyacrylamide (ApllyChem) gel and overlaid with a 0.5% (w/v) low melting agarose solution. The second-dimension separation was carried out vertically in a Protean Plus Dodeca Cell (Bio-Rad), at 3 W/gel for 30 min, followed by 200 V for 6 h, at 20 °C [35]. Gels were stained with Flamingo fluorescence stain (Bio-Rad) and the images were acquired with Molecular Imager FX (Bio-Rad).

### Gel analysis

Gel images were imported into PDQuest™ 8.0, and the spots were detected and matched through the entire match set. After automated matching, according to the parameters chosen, manual spot detection and matching were performed to confirm the results obtained using software automated functions. After matching, gel images were normalized using the “Local Regression Model” algorithm, available in PDQuest™ 8.0.

### Semi-denaturing gel electrophoresis

Twenty microliter CSF were subjected to electrophoresis in a 15% acrylamide gel without SDS. Samples were in sample buffer without SDS or reducing agent and loaded into the gel without denaturation at high temperature. TGS electrophoresis buffer was used to perform a semi-denaturing electrophoresis assay. Proteins were electroblotted onto PVDF membrane in a Semi-dry iBlot system (Invitrogen). After blocking, immunodetection was performed using anti-human TTR (DAKO) diluted (1/200) with 2.5% skimmed milk for 1 h. After washing with PBST, followed by incubation with sheep anti-rabbit immunoglobulins–HRP conjugated (Pierce; 1:5000 dilution), TTR was visualized using the enhanced chemiluminescence method (ECL, GE Healthcare). Densitometry and quantitative analysis of images were performed using Image J (NIH) software. Total conformers % was calculated by dividing the densitometry levels for the conformer fraction by the total TTR immunoreactivity (conformer, dimer and monomer).

### Protein identification and validation

Spots of interest were excised from stained gels with an automated picking using EXQuest™ Spot Cutter (Bio-Rad). Spots were destained with a solution of 15 mM K_4_Fe(CN)_6_ (potassium ferrocyanide) (Sigma) and 50 mM Na_2_S_2_O_3_ (sodium thiosulfate) (Sigma), washed with water, dehydrated using a speed vac, and incubated overnight with 10 μL trypsin (Roche) (10 mg/mL in 10 mM ammonium bicarbonate (Fluka)). Peptides were extracted with 30%, 50% and 98% acetonitrile in 1% formic acid, pooled, dried by rotary evaporation under vacuum, and resuspended in 2% acetonitrile and 0.1% formic acid.

Protein identification experiments were carried out on a hybrid quadrupole/linear ion-trap mass spectrometer (4000 QTrap; Applied Biosystems/MDS Sciex) using an electrospray source and a dual gradient pump (Ultimate 3000; Dionex). The mass spectrometer was programmed for information dependent acquisition (IDA) scanning full spectra, followed by an enhanced resolution scan to determine the ion charge states and set the appropriate collision energy for fragmentation. The IDA cycle was programmed to perform 8 MS/MS on multiple charged ions (+ 1 to + 4) and perform two repeats before adding ions to the exclusion list for 60 s (mass spectrometer operated by Analyst 1.4.1). Peptides were eluted into the mass spectrometer with a binary gradient (300 nL/min 2% acetonitrile, 0.1% formic acid to 98% acetonitrile, 0.1% formic acid in a multiple-step gradient for 50 min) (Ultimate 3000, Dionex), using a nano-electrospray source [36, 37]. Peptide identification was performed with Protein Pilot software (*v5*, Sciex) or Mascot against the SwissProt database. Positive identifications were considered when peptides had a probability score above 95%. In Protein Pilot, positive identifications were considered when the protein score was above 1.3 (95%) [37] or 2.0 (99%).

For validation, the following commercial ELISA kits were used; Agrin (ab216945; Abcam), Alpha-1-antichymotrypsin (ab157706; Abcam), Angiotensinogen (RAB1021; Sigma-Millipore), Apolipoprotein E (ab108813; Abcam), Gelsolin (ABK1-E1725; Abyntek), Prostaglandin-H2 D isomerase (10007684; Cayman Chemical) and Retinol-Binding Protein 4 (DRB400; R&D Systems). The following mean coefficient of variation (CV%) for intra/inter assay were reported by manufacturer’s of the ELISA kits: Agrin (CV; 4.2/5.7%), Alpha-1-antichymotrypsin (CV; < 10/10%), Angiotensinogen (CV; < 10/12%), Apolipoprotein E (CV; 4.4/9.7%), Gelsolin (CV; < 10/12%), Prostaglandin-H2 D isomerase (CV; < 7.2/< 10.5%), Rbp4 (CV; < 8.1/8.6%). Each kit was performed according to the manufacturer’s instructions. For each assay, a standard curve was generated, and each sample was analysed in duplicate. Importantly, the set of samples used in the validation study were from a different group of patients, and there was no overlap with the initial population used for the proteomics study. The use of independent groups of samples ensures that the variable signatures are heterogenous enough to avoid a biased effect towards what was observed in the discovery group [38]. Some of the serum and CSF samples were from the same patients: this was the case of 52% of the serum samples from patients with RRMS, 90% of the serum samples from patients with non-inflammatory diseases of the CNS and 88% of the serum samples from patients with other inflammatory diseases of the CNS.

### Data analysis

A combination of univariate (Student’s *t* test) and multivariate (Principal Component Analysis [PCA] and Linear Discriminant Analysis [LDA]) statistical analysis was employed to find the protein spots that could discriminate the groups under study. Relative volumes/intensities of matched protein spots were exported from PDQuest™ 8.0 and all the analyses were performed using Excel and SPSS (Statistical Package for the Social Sciences).

Due to the heterogeneity present in 2D-SDS-PAGE, the data set was sieved out and only spots that were present in at least 50% of the samples belonging to the three groups analysed (RRMS, other inflammatory diseases of the CNS or non-inflammatory diseases of the CNS) were considered for further investigation. The proteomics data set obtained also contains missing values that had to be imputed before multivariate analysis, which requires complete data [39]. For univariate methods, missing values are also problematic, because they reduce the number of replicates for certain spots and thus the statistical power of the test [40]. In most cases, missing values occur due to technical problems during the electrophoretic process (pH variations in the running buffer, incomplete or over-focusing in the first dimension, bad transfer from first to second dimension, gel-to-gel variations in staining or local differences in protein migration on the gel, high background noise, insufficient resolution of spots or faulty detection and separation of nearby spots) [40]. Therefore, for each missing spot we used the quantification of the average intensity of the same spot across all experiments in the same group [40].

Statistical analysis of the results obtained in the validation studies was performed using R programming language (version 3.6.2). Hierarchical Cluster Analysis (HCA) was constructed using the Ward method upon Euclidean distances and used to evaluate the relationship between protein content and the individuals. PCA was employed to identify the vectors along which variation is maximal, providing information on patients’ response in terms of proteins abundance. LDA was used to maximize the separation between the patients with RRMS and other diseases (other inflammatory and non-inflammatory diseases). The above-mentioned techniques were performed on the scaled data using *FactomineR*, *factoextra* and *MASS* packages in R.

## Results

### CSF samples analysis by 2D electrophoresis

To investigate the differences between the proteome of CSF samples obtained from patients diagnosed with RRMS and other neurological (inflammatory and non-inflammatory) diseases of the CNS, a 2D-electrophoresis approach was used. A representative gel obtained for human CSF samples is shown in Additional file 1: Fig. S1. Analysis of the gel images using PDQuest™ 8.0 identified more than 300 spots per sample. A combination of two different methods of statistical analysis (univariate and multivariate) was employed to find protein spots that could discriminate the groups under study in the data set created with a dimension of 138 samples per 223 variables (intensities of protein spots). Using univariate analysis with Student’s *t* test, we first compared the relative volume of protein spots, tested individually, in the following groups of samples: (i) RRMS vs. other inflammatory diseases of the CNS, (ii) RRMS vs. non-inflammatory diseases of the CNS, and (iii) RRMS vs. other inflammatory diseases and non-inflammatory diseases of CNS as a single group (*p* values ≤ 0.05 were considered statistically significant). Using this test, significant differences were found in the relative volume of several protein spots between groups (Additional file 2: Fig. S2).

**Fig. 2:**
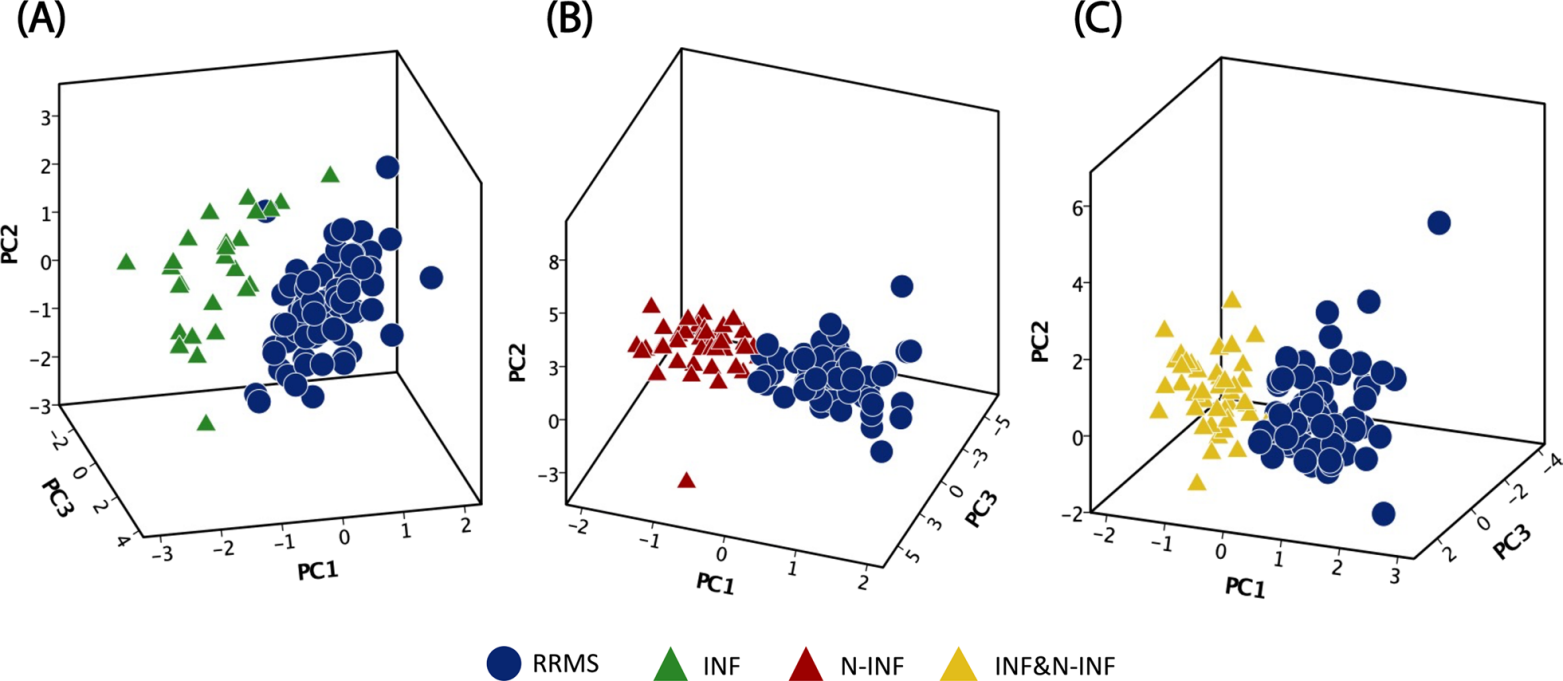
3D representation of the main principal components obtained after PCA for (**A**) the 68 protein spots found to distinguish CSF samples from patients with Relapse–remitting multiple sclerosis (RRMS) and other inflammatory diseases of the CNS (INF), using the Student’s *t*-test; (**B**) the 52 protein spots found to distinguish samples from patients with Relapse–remitting multiple sclerosis (RRMS) and non-inflammatory diseases of the CNS (N-INF), as determined by the Student’s *t*-test; and (**C**) the 52 protein spots found to distinguish samples obtained from patients with Relapse–Remitting multiple sclerosis (RRMS) vs. other inflammatory and non-inflammatory diseases of the CNS (INF and N-INF), as determined by the Student’s *t* test

Considering the protein spots obtained with the previous test, multivariate analysis (using Principal Component Analysis (PCA) and Linear Discriminant Analysis (LDA)) was used to determine whether specific patterns were present, to assist in selecting spots that could better discriminate the groups and classify the samples in their original groups. PCA is a multivariate pattern recognition method that highlights similarities and differences of data. This method represents the objects (groups of samples) described by the original variables (protein intensities) in a new reference system characterized by new variables called principal components (PC) [41]. PCA allows the identification of groups of samples and provides a size reduction of the data set, since only the relevant principal components are preserved [42]. LDA classifies the samples present in the data set by building functions to characterize the groups, and measures the degree of success of the classification model created [42]. This methodology allowed the identification of a set of protein spots that are differentially expressed in CSF samples from patients with RRMS and with other inflammatory and/or non-inflammatory diseases of the CNS.

i. RRMS vs. other inflammatory diseases of the CNS
  Considering the 68 differentially expressed protein spots identified by univariate analysis for RRMS vs. other inflammatory diseases of CNS, PCA analysis shows that the first three PC’s could account for 25% of the total variance contained in the data set (Additional file 7: Table S1) and allowed the identification of two groups (Fig. 2A). To find a protein pattern that discriminates the samples into groups, it is desirable that the number of spots is minimal. To reduce the number of protein spots that could be used to distinguish RRMS from other inflammatory diseases of the CNS, we analyzed the variables with the highest loadings (the weights of the original variables on each PC) in the main PCs. According to the factor matrix generated by SPSS software, the spots with loadings above 0.5 in the first PC (the main PC according to the scree plot given by the analysis) were considered for further analysis. Thus, a sub-data set formed by nine protein spots (1502, 1703, 3004, 3006, 3103, 4101, 7404, 7811 e 8301) was created, and LDA allowed correct discrimination of 96.9% of the samples tested, with cross-validation of 95.8% (Table 3).
ii. RRMS vs. non-inflammatory diseases of the CNS
  In parallel, PCA analysis was performed considering the intensity of protein spots found to distinguish RRMS from non-inflammatory diseases of the CNS. The results show that the first three PCs account for 23% of the total variance (Additional file 7: Table S2), which allows clustering the samples in two groups (Fig. 2B). Considering the spots with the highest loading in the first PC (5005, 7404, 7807, 7811 and 8006), a correct classification of 85.6%, with a cross-validation of 85.6% was obtained with LDA (Table 4).
iii. RRMS vs. other inflammatory diseases of CNS and non-inflammatory diseases of the CNS as a single group
  PCA was performed as well using the set of spots found to distinguish RRMS vs. other inflammatory diseases of the CNS and non-inflammatory diseases of the CNS, as determined by the Student’s *t* test. Two sample groups were formed (Fig. 2C) and considering the spots with the highest loadings, 81.9% of the samples were correctly classified, with cross-validation of 81.9% as determined with LDA (Table 5).

**Table 3:** Sample classification after LDA to discriminate between CSF samples from patients with Relapse–remitting multiple sclerosis (RRMS) and other inflammatory diseases of the CNS (INF)

	Disease	Predicted Group Membership^b.c^		Total
		RRMS	INF	
Original				
Count	RRMS	68	1	69
	INF	2	25	27
%	RRMS	98.6	1.4	100
	INF	7.4	92.6	100
Cross-validated^a^				
Count	RRMS	67	2	69
	INF	2	25	27
%	RRMS	97.1	2.9	100
	INF	7.4	92.6	100

^a^Cross-validation is done only for those sets of samples analyzed. In cross-validation, each sample is classified by the functions derived from all samples other the one under analysis

^b^96.9% of original grouped samples correctly classified

^c^95.8% of cross-validated grouped samples correctly classified

**Table 4:** Sample classification after LDA to discriminate between CSF samples from patients with Relapse–remitting multiple sclerosis (RRMS) and non-inflammatory diseases of the CNS (N-INF) after data reduction with PCA

	Disease	Predicted Group Membership^b.c^		Total
		RRMS	N-INF	
Original				
Count	RRMS	66	3	69
	N-INF	13	29	42
%	RRMS	95.7	4.3	100
	N-INF	31.0	69.0	100
Cross-validated^a^				
Count	RRMS	66	3	69
	N-INF	13	29	42
%	RRMS	95.7	4.3	100
	N-INF	31.0	69.0	100

^a^Cross-validation is done only for those sets of samples analyzed. In cross-validation, each sample is classified by the functions derived from all samples other the one under analysis

^b^85.6% of original grouped samples correctly classified

^c^85.6% of cross-validated grouped samples correctly classified

**Table 5:** Sample classification after LDA to discriminate between CSF samples from patients with Relapse–remitting multiple sclerosis (RRMS) vs. other diseases (inflammatory and non-inflammatory) of the CNS (INF and N-INF) after data reduction with PCA.

	Disease	Predicted Group Membership^b.c^		Total
		RRMS	INF and N-INF	
Original				
Count	RRMS	55	14	69
	INF and N-INF	11	58	69
%	RRMS	79.7	20.3	100
	INF and N-INF	15.9	84.1	100
Cross-validated^a^				
Count	RRMS	55	14	69
	INF and N-INF	11	58	69
%	RRMS	79.7	20.3	100
	INF and N-INF	15.9	84.1	100

^a^Cross-validation is done only for those sets of samples analyzed. In cross-validation, each sample is classified by the functions derived from all samples other the one under analysis

^b^81.9% of original grouped samples correctly classified

^c^81.9% of cross-validated grouped samples correctly classified

### Identification of the protein spots selected by LC/MS/MS

#### RRMS vs. other inflammatory diseases of the CNS

**Fig. 3:**
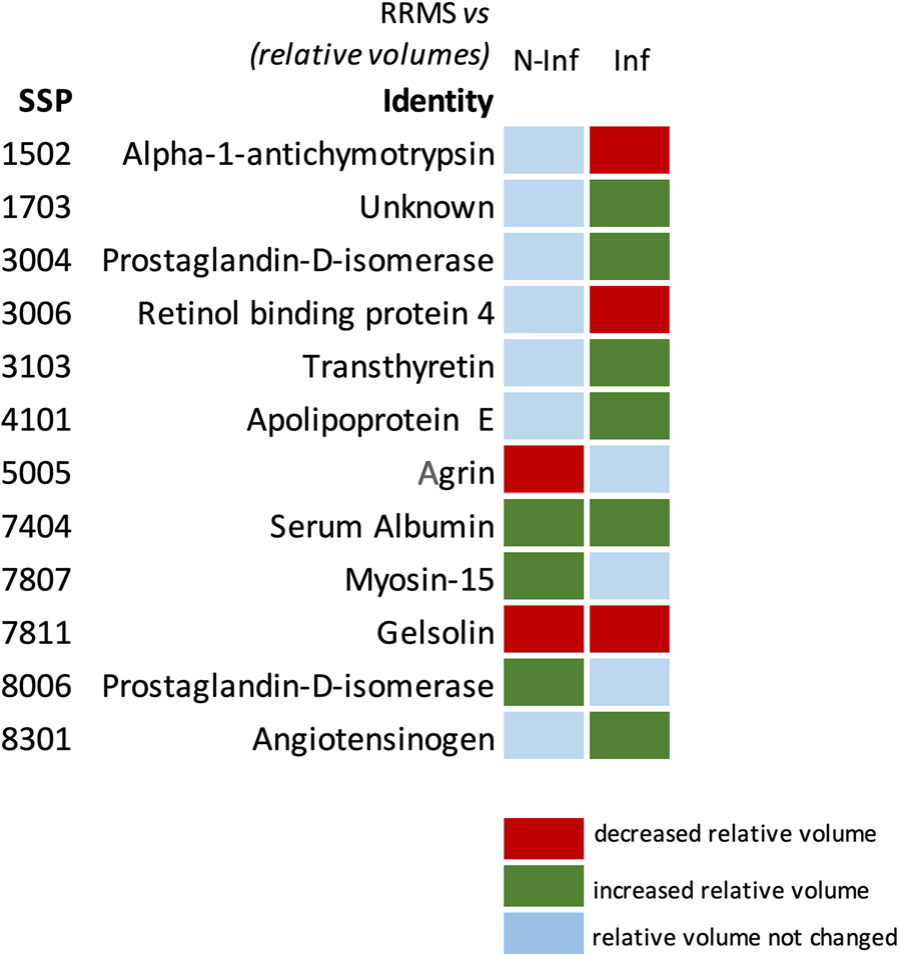
Map summarizing the protein spots that are differentially expressed between Relapse–remitting multiple sclerosis (RRMS), non-inflammatory (N-INF) and other inflammatory (INF) diseases of the CNS

From the results obtained, the protein spots that best discriminate RRMS from other CNS inflammatory diseases are 1502, 1703, 3004, 3006, 3103, 4101, 7404, 7811 and 8301. These spots were analyzed by mass spectrometry, and seven of them were identified as: Alpha-1-antichymotrypsin (1502), Prostaglandin-H2 D isomerase (3004), Retinol binding protein 4 (Rbp4; 3006), Transthyretin (TTR; 3103), Apolipoprotein E (ApoE; 4101), Gelsolin (7811) and Angiotensinogen (8301) (Fig. 3 and Additional file 7: Table S3). One of the spots was not identified due to low mass spectrometry signal intensity, insufficient number of peptides, or low protein stability.

LDA is used when classes are known a priori (RRMS, inflammatory diseases of the CNS and non-inflammatory diseases of the CNS). The LDA algorithm starts by finding directions that maximize the separation between classes, then use these directions to predict the class of individuals. These directions - linear discriminants - are a linear combinations of predictor variables. LDA for the seven protein spots identified allows a correct classification of 91.7%, with cross-validation of 87.5% (Table 6). The protein spot number 7404 was mainly identified as Serum albumin, one of the most abundant proteins in human CSF which, therefore, cannot be considered to characterize RRMS. Further studies are needed to investigate the post-translational modification(s) present in this spot. When LDA was performed with the remaining protein spots identified, a percentage of 85.4 for correct classification was obtained with a cross-validation of 83.3% (Table 7). Finally, LDA was used following sequential removal of protein spots with lower loading values and the results are summarized in Table 7. The differences observed for ApoE and Gelsolin allowed correct classification of 91.7% and cross-validation of 89.6% of the samples derived from patients with RRMS vs. other inflammatory diseases of the CNS.

**Table 6:** Sample classification after LDA to discriminate between CSF samples from patients with Relapse–remitting multiple sclerosis (RRMS) and other inflammatory diseases of the CNS (INF) based on the identified protein spots

	Classification results^b,c^			
	Disease	Predicted Group Membership		Total
		RRMS	INF	
Original				
Count	RRMS	68	1	69
	INF	7	20	27
%	RRMS	98.6	1.4	100
	INF	25.9	74.1	100
Cross-validated^a^				
Count	RRMS	67	2	69
	INF	10	17	27
%	RRMS	97.1	2.9	100
	INF	37.0	63.0	100

^a^Cross validation is done only for those cases in the analysis. In cross validation, each sample is classified by the functions derived from all samples other than the one under analysis

^b^91.7% of original grouped cases correctly classified

^c^87.5% of cross-validated grouped cases correctly classified

**Table 7:**
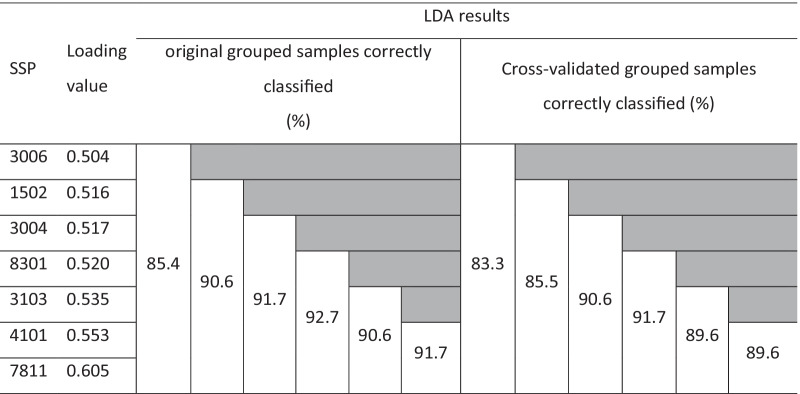
LDA results obtained by sequentially removing the protein spots with lower values of loadings in the comparison of samples from patients with Relapse-remitting multiple sclerosis (RRMS) vs. other inflammatory diseases of the CNS

#### RRMS vs. non-inflammatory diseases of the CNS

The protein spots that best distinguish RRMS from non-inflammatory diseases of CNS are 5005, 7404, 7807, 7811 and 8006. These protein spots were identified as Agrin (5005), Serum albumin (7404), Myosin-15 (7807), Gelsolin (7811) and Prostaglandin-H2-d-isomerase (PGDS; 8006) (Fig. 3 and Additional file 7: Table S4), allowing a correct sample classification and cross-validation of 85.6% (Table 4). When the protein spot identified as Serum albumin (7404) was not considered in the linear discriminant analysis a percentage of 87.4 for correct classification was obtained, with a cross-validation of 85.6% (Table 8).

**Table 8:** Sample classification after LDA to discriminate between CSF samples from patients with Relapse–remitting multiple sclerosis (RRMS) and non-inflammatory diseases of the CNS (N-INF) based on the identified protein spots

	Classification results^b,c^			
	Disease	Predicted Group Membership		Total
		RRMS	N-INF	
Original				
Count	RRMS	67	2	69
	N-INF	12	30	42
%	RRMS	97.1	2.9	100
	N-INF	28.6	71.4	100
Cross-validated^a^				
Count	RRMS	67	2	69
	N-INF	14	28	42
%	RRMS	97.1	2.9	100
	N-INF	33.3	66.7	100

^a^Cross validation is done only for those cases in the analysis. In cross validation, each sample is classified by the functions derived from all samples other than the one under analysis

^b^87.4% of original grouped cases correctly classified

^c^85.6% of cross-validated grouped cases correctly classified

#### RRMS vs. other inflammatory and non-inflammatory diseases of the CNS as a single group

The protein spots that best discriminate RRMS from the other two groups are 7001, 7404, 7811 and 8402. The spots identified correspond to EF-hand calcium-binding domain-containing protein 13 (7001), Serum albumin (7404), Gelsolin (7811) and Apolipoprotein B-100 (ApoB; 8402) (Additional file 7: Table S5), which allow the classification and cross-validation of 81.9% of the cases (Table 5). When the protein spot identified as serum albumin (7404) was not considered in the linear discriminant analysis a percentage of 83.3 for correct classification was obtained, with a cross-validation of 82.6% (Table 9).

**Table 9:** Sample classification after LDA to discriminate between CSF samples from patients with Relapse–remitting multiple sclerosis (RRMS) vs. other diseases (inflammatory and non-inflammatory) of the CNS (INF and N-INF) based on the identified protein spots

	Classification results^b,c^			
	Disease	Predicted Group Membership		Total
		RRMS	INF and N-INF	
Original				
Count	RRMS	57	12	69
	INF and N-INF	11	58	69
%	RRMS	82.6	17.4	100
	INF	15.9	84.1	100
Cross-validated^a^				
Count	RRMS	56	13	69
	INF and N-INF	11	58	69
%	RRMS	81.2	18.8	100
	INF & N-INF	15.9	84.1	100

^a^Cross validation is done only for those cases in the analysis. In cross validation, each sample is classified by the functions derived from all samples other than the one under analysis

^b^83.3% of original grouped cases correctly classified

^c^82.6% of cross-validated grouped cases correctly classified

All peptide sequences used for protein identification in all groups are described in Additional file 7: Table S6.

### Validation

Next, we aimed at further validating the results obtained in the above-described proteomics studies through enzyme-linked immunosorbent assay (ELISA). Validation was performed for the proteins that were found to distinguish the CSF of (i) patients with RRMS vs. those with other inflammatory diseases of the CNS, and (ii) patients with RRMS vs. individuals with non-inflammatory diseases of the CNS. From all the spots that better distinguished the three groups of patients, we analyzed the CSF content in Alpha-1-antichymotrypsin (1502), Gelsolin (7811), Agrin (5005), PGDS (3004), Angiotensinogen (8301), ApoE (4101) and Rbp4 (3006), using commercial ELISA kits, while TTR (3103) was analyzed by Western Blot. Myosin-15 (7807) was not analyzed, since no reliable commercial antibodies are available. Whenever differences were observed between the three groups in CSF samples, the analysis was extended to the serum.

**Fig. 4:**
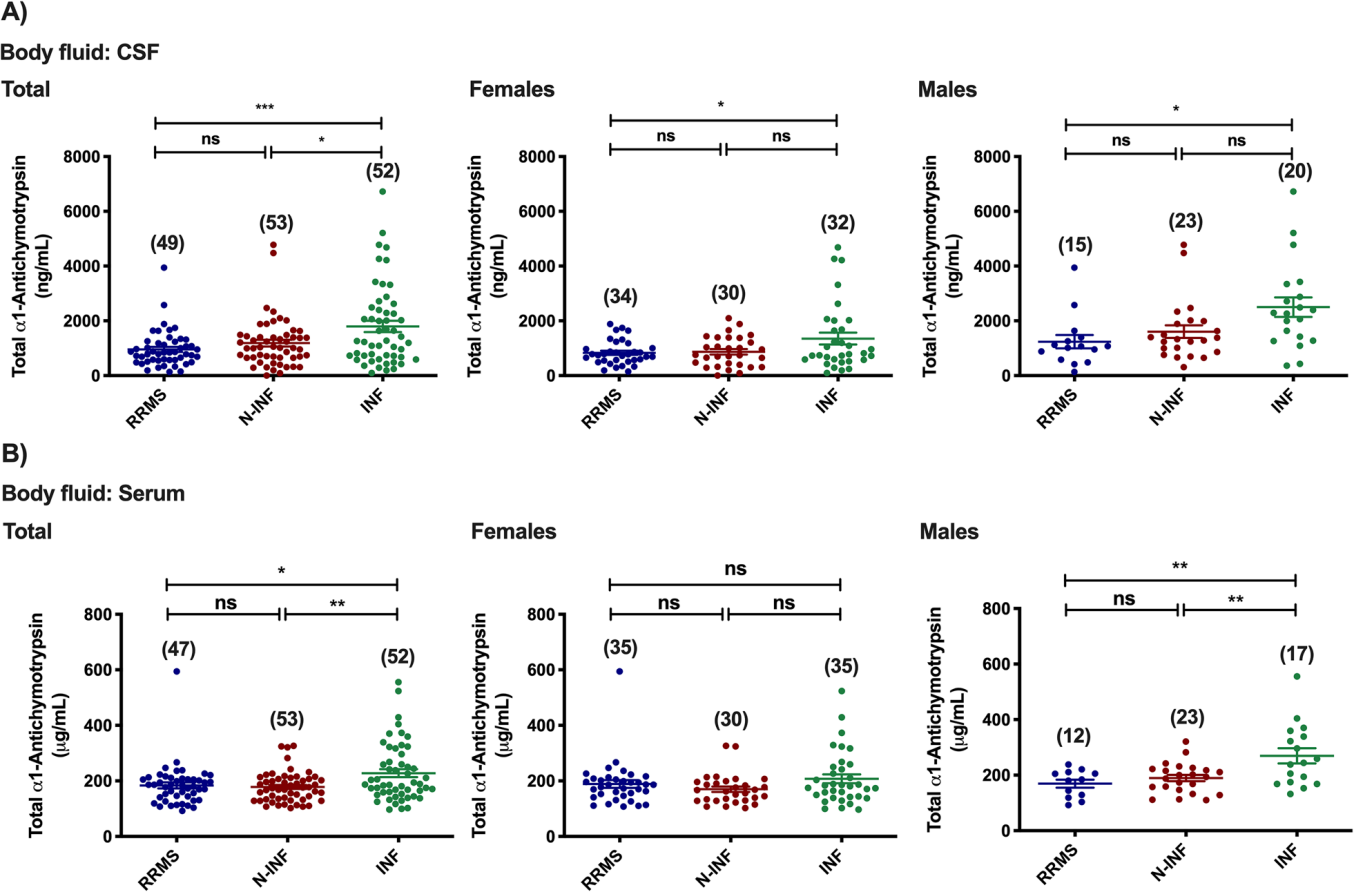
Total abundance of Alpha-1-antichymotrypsin in the CSF (**A**) and serum (**B**) of patients diagnosed with Relapse–remitting multiple sclerosis (RRMS), non-inflammatory diseases of the CNS (N-INF), and with other inflammatory diseases of the CNS (INF), and further sub-divided by gender. The results represent the mean ± SEM and statistical analysis was performed by one-way ANOVA followed by the Tukey’s multiple comparison test, comparing all the indicated conditions (ns, *p* > 0.05; *p ˂ 0.05, **p ˂ 0.01, ***p < 0.001)

In the population of patients enrolled in the study, we observed a significant decrease in the total abundance of Alpha-1-antichymotrypsin in the CSF of individuals diagnosed with RRMS and with non-inflammatory diseases of the CNS when compared with patients with inflammatory diseases of the CNS (Fig. 4A: INF [1795.03 ng/mL] vs. RRMS [956.68 ng/mL], *p* < 0.001; and N-INF [1187.46 ng/mL], *p* < 0.05). When further sub-divided by gender, females and males showed significant differences between the RRMS and other inflammatory diseases groups (Fig. 4A: RRMS [833.18 ng/mL] vs. INF [1352.95 ng/mL] (*p* < 0.05) and RRMS [1237.25 ng/mL] vs. INF [2502.37 ng/mL] (*p* < 0.05), respectively). In serum samples analyzed together, Alpha-1-antichymotrypsin protein levels in patients diagnosed with RRMS and with non-inflammatory diseases of the CNS were also significantly lower than the levels of the protein detected in samples from individuals diagnosed with other inflammatory diseases of the CNS (Fig. 4B: INF [227.96 μg/mL] vs. RRMS [183.93 μg/mL] vs. *p* < 0.05; and N-INF [178.43 μg/mL]). Similar results were obtained when the analysis was limited to samples obtained from male patients (Fig. 4B: INF [269.71 μg/mL] vs. RRMS [169.50 μg/mL], *p* < 0.01); and N-INF [189.36 μg/mL], *p* < 0.01), but not from the female group.

**Fig. 5:**
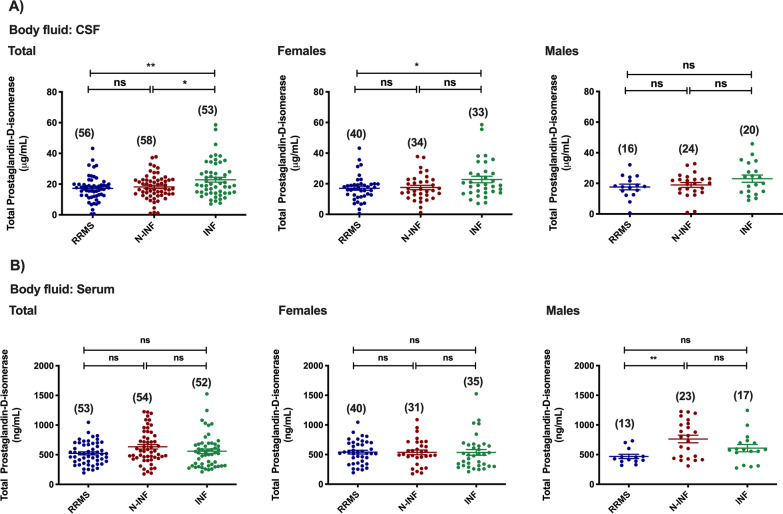
Total abundance of Prostaglandin H2-D-isomerase in the CSF (**A**) and serum (**B**) of patients diagnosed with Relapse–remitting multiple sclerosis (RRMS), non-inflammatory diseases of the CNS (N-INF), and with other inflammatory diseases of the CNS (INF), and further sub-divided by gender. The results represent the mean ± SEM and statistical analysis was performed by one-way ANOVA followed by the Tukey’s multiple comparison test, comparing all the indicated conditions (ns, *p* > 0.05; *p ˂ 0.05, **p ˂ 0.01)

For PGDS, a decrease in total protein expression was observed in the CSF of patients belonging to the RRMS and inflammatory CNS diseases groups when compared with samples from patients with inflammatory diseases of the CNS (Fig. 5A: INF [22.86 μg/mL] vs. RRMS [17.22 μg/mL], *p* < 0.01; and N-INF [18.14 μg/mL], *p* < 0.05). When stratified by gender, differences were only found between the RRMS and other inflammatory CNS diseases groups in females (Fig. 5A: INF [22.74 μg/mL] vs. RRMS [17.06 μg/mL], *p* < 0.05), whereas no alterations were detected in males in all groups (*p* > 0.05). For this analyte, a decrease in total protein abundance was only found in the serum of males with RRMS group when compared to the other non-inflammatory diseases of the CNS group (Fig. 5B: RRMS [469.46 ng/mL] vs*.* N-INF [763.17 ng/mL], *p* < 0.01).

**Fig. 6:**
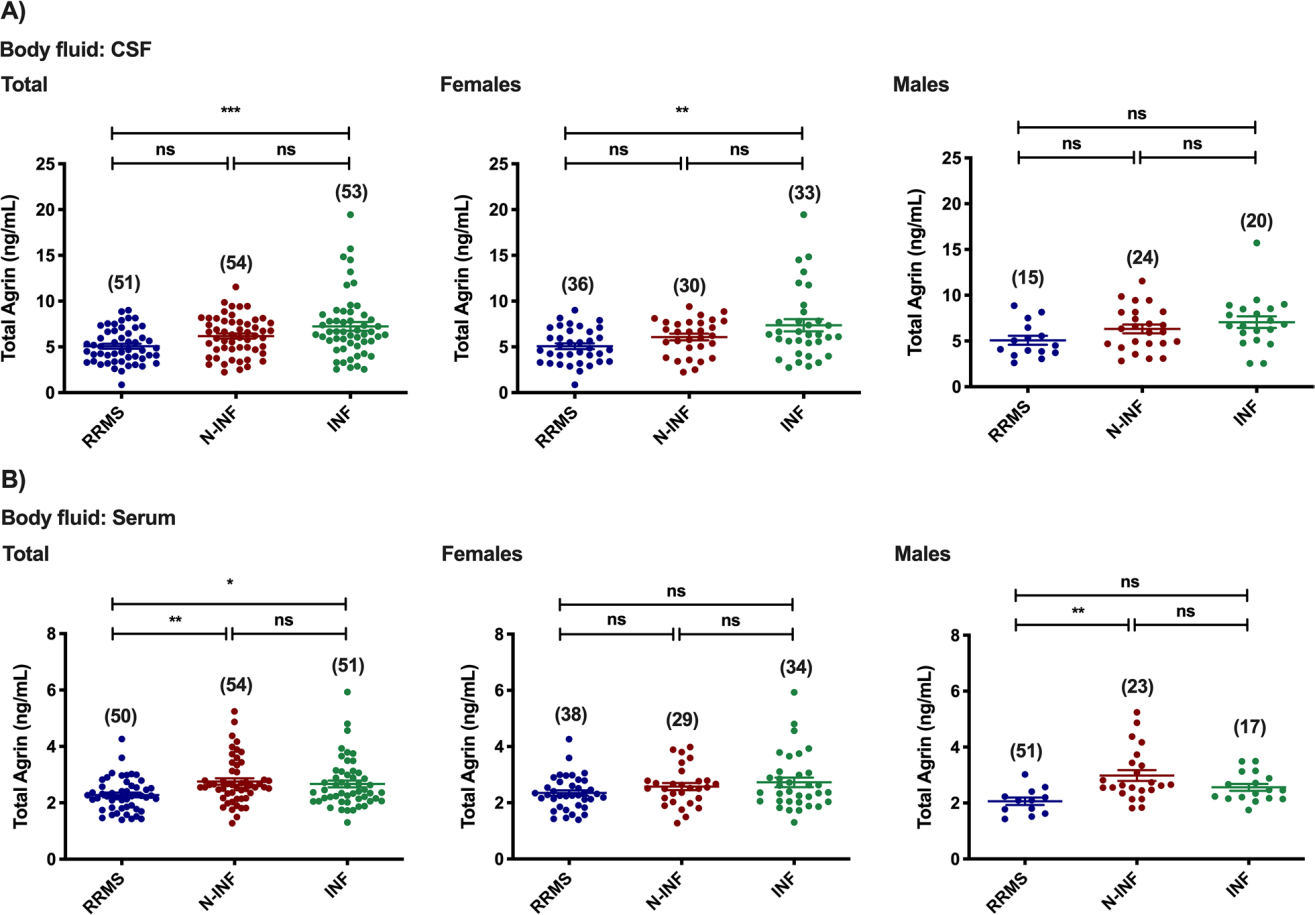
Total abundance of Agrin in the CSF (**A**) and serum (**B**) of patients diagnosed with Relapse–remitting multiple sclerosis (RRMS), non-inflammatory diseases of the CNS (N-INF), and with other inflammatory diseases of the CNS (INF), and further sub-divided by gender. The results represent the mean ± SEM and statistical analysis was performed by one-way ANOVA followed by the Tukey’s multiple comparison test, comparing all the indicated conditions (ns, *p* > 0.05; *p ˂ 0.05, **p ˂ 0.01, ***p ˂ 0.001)

Total Agrin levels in the CSF were found to be downregulated in patients diagnosed with RRMS when compared with the other inflammatory diseases of the CNS group (Fig. 6A: RRMS [5.07 ng/mL] vs. INF [7.24 ng/mL], *p* < 0.001). Similar changes were observed in females (Fig. 6A: RRMS [5.07 ng/mL] vs. N-INF [7.36 ng/mL], *p* < 0.01), but not in males (*p* > 0.05 in all experimental groups). When evaluated in the serum, RRMS patients (Fig. 6B: RRMS [2.27 ng/mL] vs. N-INF [2.75 ng/mL], *p* < 0.01; and INF [2.67 ng/mL], *p* < 0.05) showed the lowest total Agrin protein levels among the three groups. Lower levels of Agrin were still detected in the serum of RRMS male patients when compared to the group of patients with non-inflammatory diseases (Fig. 6B: RRMS [2.06 ng/mL] vs. N-INF [2.98 ng/mL], *p* < 0.01), but not in females (*p* > 0.05 in all experimental groups).

**Fig. 7:**
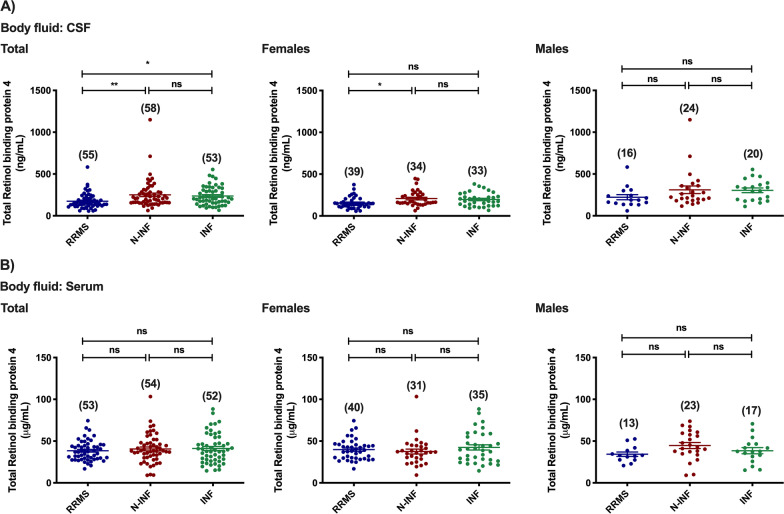
Total abundance of Rbp4 in the CSF (**A**) and serum (**B**) of patients diagnosed with Relapse–remitting multiple sclerosis (RRMS), non-inflammatory diseases of the CNS (N-INF), and with other inflammatory diseases of the CNS (INF), and further sub-divided by gender. The results represent the mean ± SEM and statistical analysis was performed by one-way ANOVA followed by the Tukey’s multiple comparison test, comparing all the indicated conditions (ns, *p* > 0.05; *p ˂ 0.05, **p ˂ 0.01)

In the case of Rbp4, lower levels of the protein were found in the CSF of patients diagnosed with RRMS when compared with samples from patients belonging to the other two groups (Fig. 7A: RRMS [174.41 ng/mL] vs. N-INF [250.65 ng/mL], *p* < 0.01; and INF [237.39 ng/mL], *p* < 0.05). Breaking it down by gender showed lower levels of the protein in RRMS female patients when compared to females belonging to the group of patients with non-inflammatory CNS diseases (Fig. 7A: RRMS [154.71 ng/mL] vs. N-INF [207.91 ng/mL], *p* < 0.05), whereas total levels of the protein were similar in male groups (*p* > 0.05 in all experimental groups). No changes were found when serum was used as the source of biological fluid in both genders (*p* > 0.05 in all experimental groups).

In contrast with all other analytes validated, we found no differences in total levels of ApoE (Additional file 3: Fig. S3) and Angiotensinogen (Additional file 4: Fig. S4), irrespectively of gender and experimental group, in the CSF of these patients (*p* > 0.05 in all experimental groups). Therefore, the levels of these proteins were not analyzed in the serum.

**Fig. 8:**
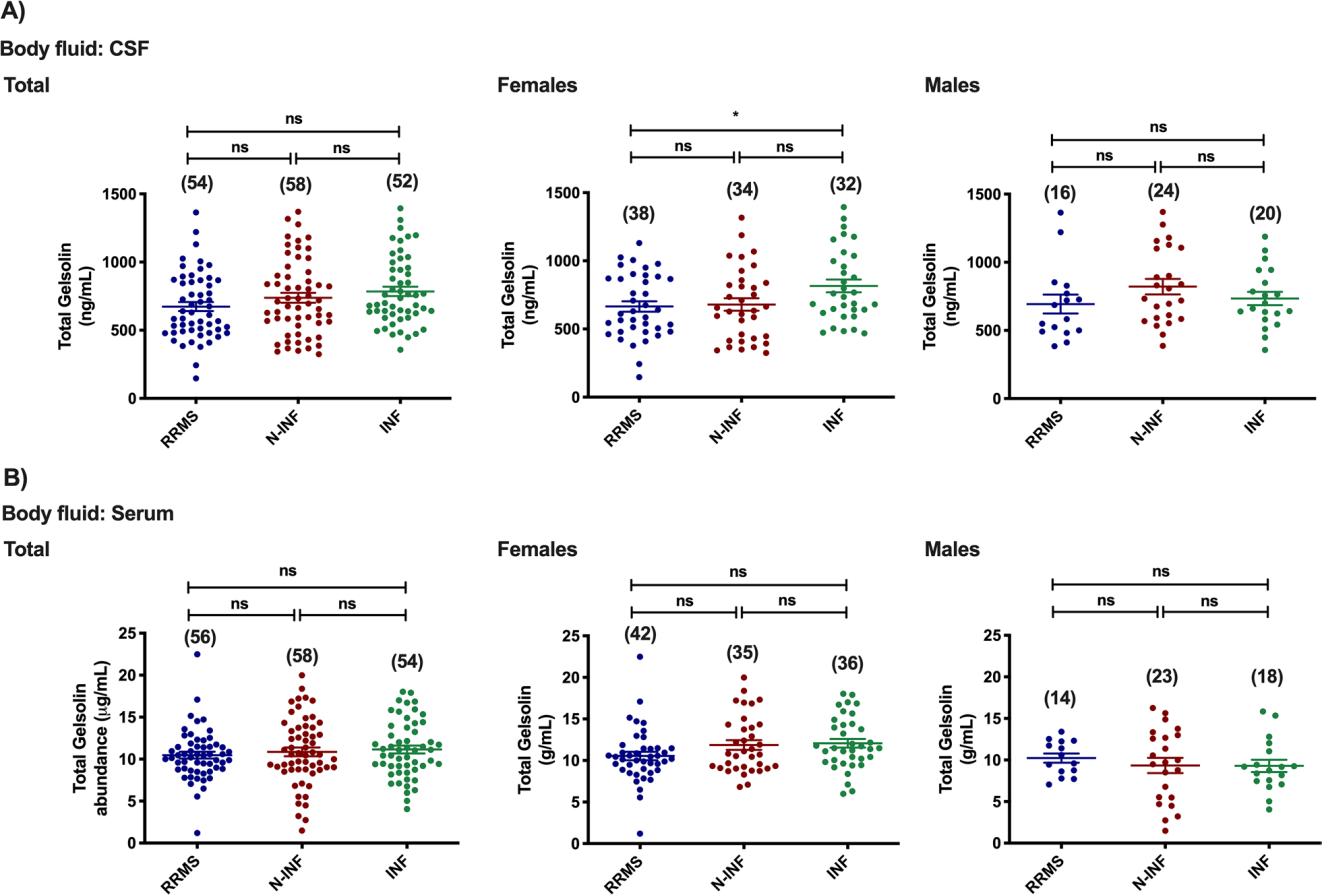
Total abundance of Gelsolin in the CSF (**A**) and serum (**B**) of patients diagnosed with Relapse–remitting multiple sclerosis (RRMS), non-inflammatory diseases of the CNS (N-INF), and with other inflammatory diseases of the CNS (INF), and further sub-divided by gender. The results represent the mean ± SEM and statistical analysis was performed by one-way ANOVA followed by the Tukey’s multiple comparison test, comparing all the indicated conditions (ns, *p* > 0.05; *p ˂ 0.05)

Total Gelsolin protein levels were found to be decreased in the CSF of RRMS female patients when compared to the group of female patients with other inflammatory CNS diseases (Fig. 8A: RRMS [665.33 ng/mL] vs. INF [816.12 ng/mL], *p* < 0.05). No differences were observed when the Gelsolin content in the CSF of all patients (males and females) belonging to the three groups was analyzed together, as well as in males. Similar results were obtained in serum samples from the three groups of patients (*p* > 0.05 in all experimental groups).

**Fig. 9:**
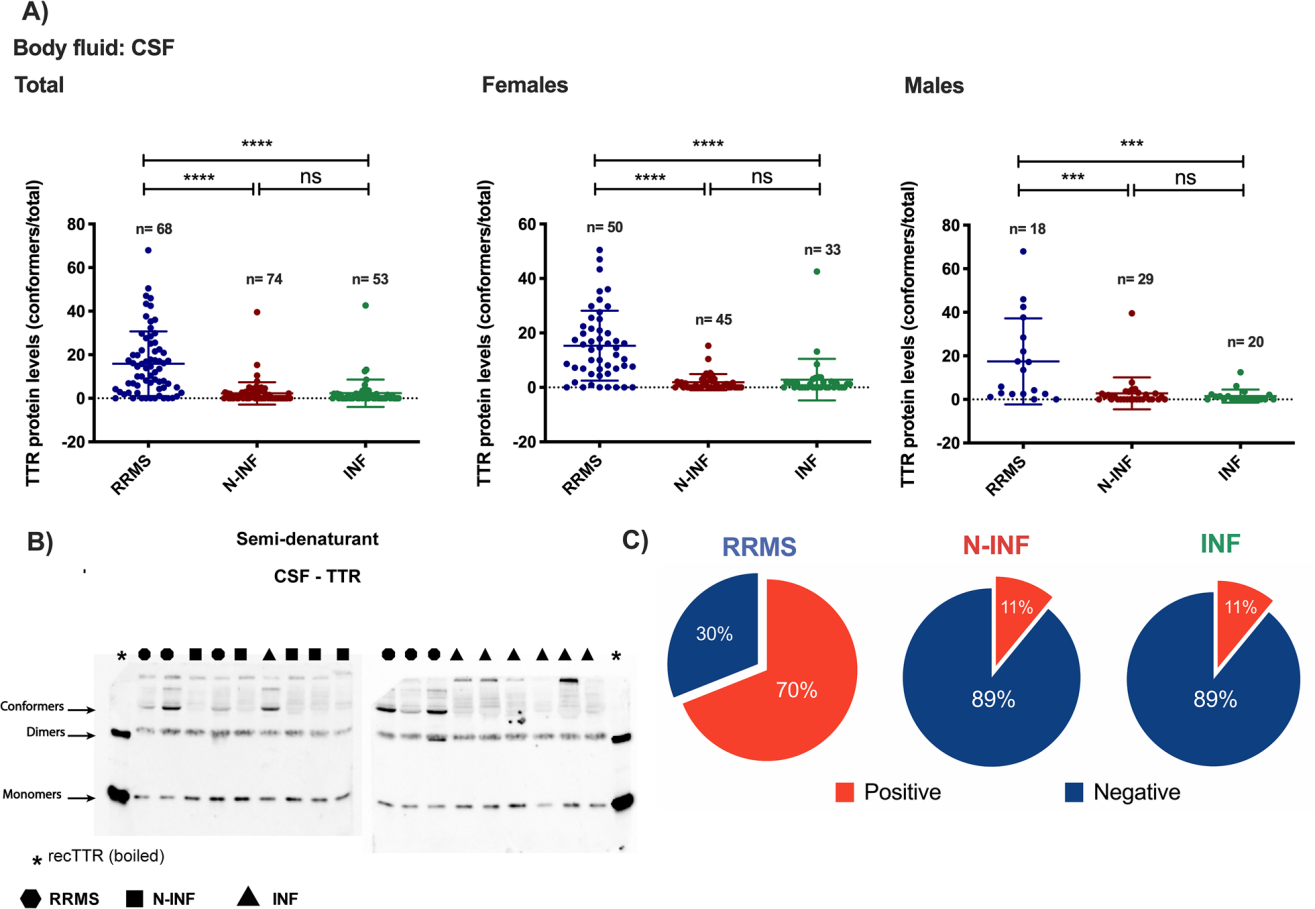
TTR profile in CSF samples from Relapse–remitting multiple sclerosis (RRMS), other inflammatory diseases of the CNS (INF) and with non-inflammatory diseases of the CNS (N-INF), analysed by western blot under semi-denaturating conditions. **A**, **B** CSF samples belonging to the three groups were subjected to PAGE followed by western blot with an anti-TTR antibody. The migration of TTR monomers, dimers and conformers is shown in the image. **A** Fraction of the total TTR immunoreactivity in the conformer form in CSF samples from the three groups. **C** Percentage of samples from the three groups showing TTR conformers (ns, *p* > 0.05; ***p < 0.001; ****p < 0.0001)

TTR was one of the proteins identified by mass-spectrometry analysis, and its aggregation was previously proposed to be associated with MS pathology [43]. To further validate our findings, we analyzed total TTR levels on the CSF of patients from all experimental groups. We did not observe differences in total TTR protein abundance when evaluated using ELISA (data not shown). However, differences were observed in the pattern of TTR migration when analyzed by polyacrylamide gel electrophoresis (PAGE) under semi-denaturing conditions. A significant increase in high molecular weight TTR (conformers) species was observed in the CSF of patients with RRMS when compared to the other two experimental groups, where these forms are barely observed (Fig. 9A, B: RRMS [15.89%] vs. non/INF [2.25/2.36%], *p* < 0.0001]). The differences observed when all patients were analyzed together are completely transposed to females (Fig. 9A, B: RRMS [15.33%] vs. non/INF [1.91/2.86%]; *p* < 0.0001), and males (Fig. 9A, B: RRMS [17.46%] vs. non/INF [2.78/1.53%]; *p* < 0.0001). In an attempt to classify a patient as positive for TTR conformers, which may help to better clarify the etiology of the disease, a threshold of 5% was used to classify as conformer positive. This approach was used to determine the percentage of patients that are positive for this marker. Figure 9C clearly shows that most patients belonging to the RRMS group (70%), show ‘positive’ immunoreactivity for this marker, whereas roughly 11% of patients from the other two groups in this study show this kind of molecular signature. These species are believed to be composed of aggregated and oxidized TTR protein and were not detected in the serum of RRMS patients [43]. Therefore, we have not analyzed alterations in TTR in the serum of RRMS patients.

**Fig. 10:**
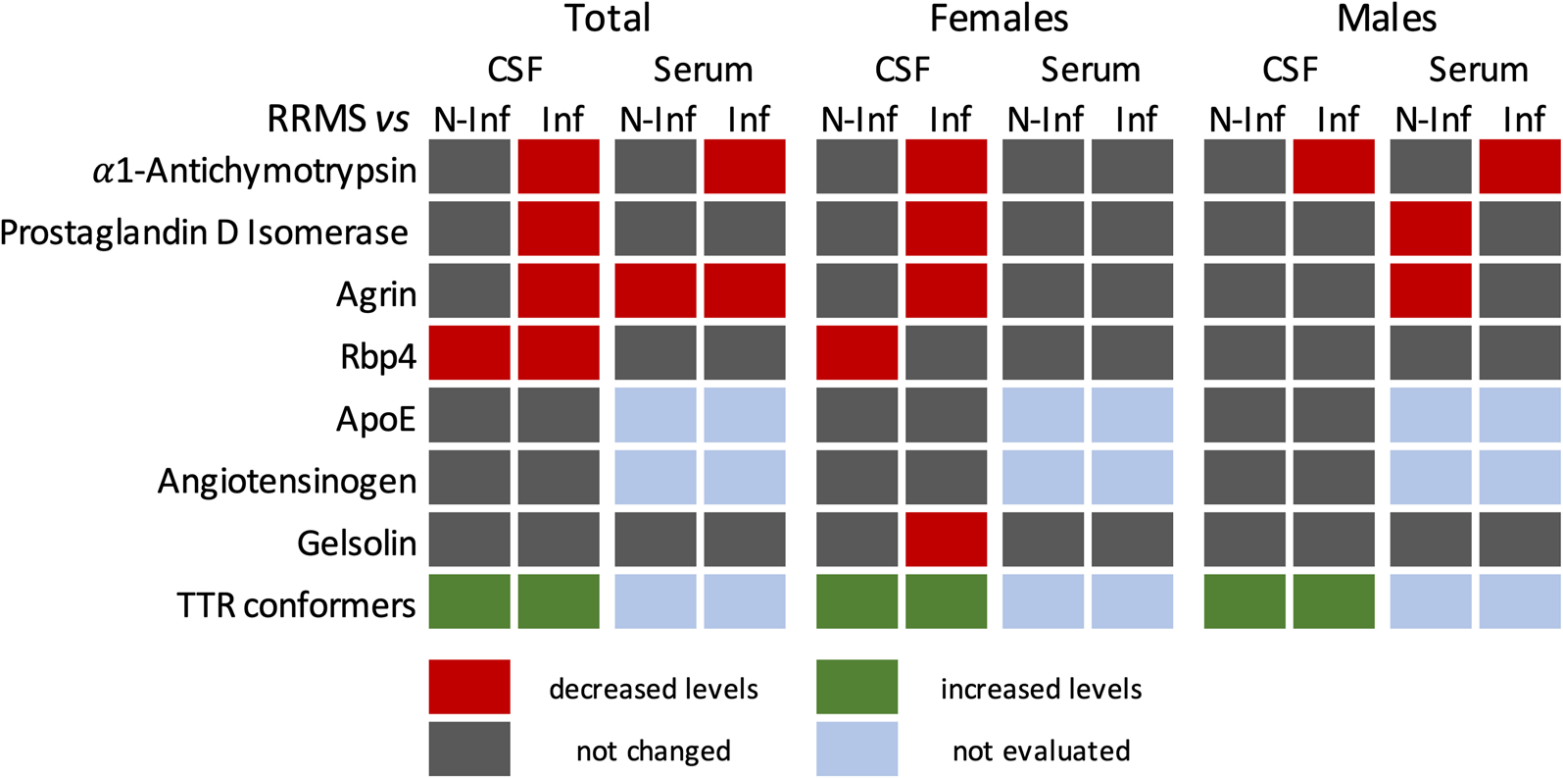
Map summarizing the changes of each individual protein comparing their total abundance in the Relapse–remitting multiple sclerosis (RRMS) group with the non-inflammatory (N-INF) and other inflammatory (INF) diseases of CNS groups, respectively

Total changes observed in all proteins tested are summarized in Fig. 10.

### Analysis based on the validated proteins in CSF samples

Considering the results above, the proteins Alpha-1-antichymotrypsin, PGDS, RBP4, TTR, ApoE, Gelsolin, Agrin, and Angiotensinogen were identified as having an impact on the discrimination between patients with RRMS and inflammatory/non-inflammatory diseases of the CNS. HCA of the results using the data obtained for these eight proteins in the validation studies with CSF samples are represented by a dendrogram in Additional file 5: Fig. S5, illustrating the relationship between the three groups of patients according to their similarity in terms of protein abundance. There is a cluster mainly composed of RRMS patients (dark blue cluster) on the dendrogram, suggesting a common pattern. The remaining groups of this dendrogram do not seem to be associated with any specific classes, except for the cluster highlighted in green, which is constituted mainly by subjects with other inflammatory diseases of the CNS.

The eigenvalues were evaluated to understand how much information was retained on the first principal components. Additional file 7: Table S7 shows the eigenvalues for all PCs, allowing to determine the number of principal components to be considered. On the first two components, approximately 47% of information is retained. With the third PC, ca. 60% of information variability is recovered.

The loadings of a PCA are the weights for each original variable when calculating the principal components and the larger the contribution of the loadings, the larger is the magnitude of the vectors in the biplot. In the latter representation of PCA, vectors that have similar direction correspond to proteins that have similar response profiles in the patients. PCA loadings for CSF scaled data are shown in Additional file 7: Table S8. The results show that Alpha-1-antichymotrypsin, PGDS, Rbp4 and Agrin have a high impact on PC1, and PGDS, Rbp4 and ApoE on PC2. Along the third component (PC3), TTR and Angiotensinogen have the highest impact. For a visual interpretation of the results, three different biplots, representing both loadings and scores, were constructed: (A) for PC1 and PC2, (B) for PC1 and PC3 and (C) for PC2and PC3 (Additional file 6: Fig. S6). In the biplots, and especially in the one represented in Additional file 6: Fig. S6B, it is noteworthy that the patients with RRMS are mainly characterized by increased TTR protein (see also Additional file 7: Table S8), resulting in a visual separation of RRMS individuals to the left side of the biplot. In the remaining biplots (B and C) it is also clear the TTR influence in RRMS patients, but the rest of the patients (INF and N-INF) do not show any particular behavior regarding the proteins analyzed. Overall, these results are in accordance with the analysis presented in Additional file 5: Fig. S5, where a cluster mainly composed of RRMS patients is visible.

The LDA results show that the RRMS group is separated from the others, while the other two classes (other inflammatory diseases and non-inflammatory diseases of the CNS) are slightly overlapping. The model accuracy, computed with a ratio test/training set of 25%, attains 79% accuracy in the separation between RRMS and the other two groups.

Finally, to understand (i) the HCA results, where RRMS patients were all in the same cluster, (ii) the PCA results, where the TTR protein had an enormous influence on RRMS individuals and (iii) the LDA results where the RRMS class is separated from the others, the means of the clusters formed in HCA (Additional file 5: Fig. S5) were calculated and are presented in Additional file 7: Table S9. The means presented in the latter table confirm the weight that proteins have in each cluster. In fact, TTR protein dominates the cluster mainly composed of RRMS individuals, highlighting its importance in the characterization of RRMS patients.

## Discussion

The main objective of this work was to identify and validate biomarkers in human CSF samples with clinical utility for the diagnosis of RRMS. It is important to find biomarkers that allow distinguishing RRMS from other inflammatory and non-inflammatory diseases of the CNS, which is difficult to perform in the clinical practice. For this reason, among others, biological samples form healthy control subjects were not included in the study. We focused on the set of proteins that best discriminates samples from (i) RRMS vs. other inflammatory diseases of the CNS, (ii) RRMS vs. non-inflammatory diseases of the CNS and (iii) RRMS vs. other inflammatory and non-inflammatory diseases of the CNS as a single group. Remarkably, statistical analysis of the results obtained in the 2D gels showed a set of protein spots that allowed a correct discrimination of 99.3% of the CSF samples from patients with RRMS vs. other inflammatory and non-inflammatory diseases of the CNS, with a cross-validation of 91.3%. It was not the aim of the present study to establish a certain threshold for protein abundance that will allow determining whether a given patient is likely to have MS.

Analysis of the protein spots of interest allowed the identification of seven proteins that are differentially expressed in CSF samples from RRMS when compared with other inflammatory diseases of the CNS, as determined by 2D-PAGE: Alpha-1-antichymotrypsin, PGDS, Rbp4, TTR, ApoE, Gelsolin and Angiotensinogen. A different set of spots distinguished CSF samples from patients with RRMS patients and with non-inflammatory diseases of the CNS: Agrin, Serum albumin, Myosin-15, PGDS and Gelsolin. Finally, EF-hand calcium-binding domain-containing protein 13, Serum albumin, Gelsolin and ApoB allowed the discrimination between samples from RRMS patients and the other two groups considered together. This is the first time that these sets of proteins are shown to distinguish CSF samples for RRMS, other inflammatory and non-inflammatory diseases of the CNS patients.

The most striking alteration in the CSF proteome in RRMS was the oligomerization of TTR in high molecular weight species (conformers) in about 70% of the samples analyzed, while in the other two groups it was limited to 11%. In a previous study, the oligomerization of TTR was associated with increased levels of sulfydration (–SH) and sulfonation (–SO3H). Since cerebral TTR is a major thyroxine (T4) carrier, it is noteworthy to mention that oxidative modifications in CSF TTR are accompanied by a downregulation in the levels of free T4 [43], suggesting that oxidative posttranslational modifications in the protein alter the capacity to act as a carrier of the hormone. In contrast with the results obtained in the present work, previous studies reported an upregulation, downregulation or no alterations in TTR protein levels in the CSF of patients with MS [20, 23, 43–46]. The differential results reflect the diversity of approaches used in previous studies to assess the TTR alterations in the CSF of MS patients and shows the relevance of using methodologies that identify posttranslational modifications of the protein, or those that can distinguish different proteoforms, such as 2D-PAGE. Other posttranslational modifications in CSF proteins were found to be altered in MS, including glycosylation [47, 48], glutathionylation [49] and proteolysis [50]. Importantly, we did not observe oligomerization of TTR in the serum of patients diagnosed with RRMS (data not shown). These results suggest that this particular alteration in TTR occurs specifically in the CNS, and that passive leakage through the brain–blood barrier (BBB) does not contribute to the observed changes in the concentration of the analytes in the CSF and serum.

Rbp4 protein levels were decreased in the CSF of patients with RRMS when compared with the results obtained in samples from patients with other inflammatory diseases of the CNS, both the proteomics analysis and in the ELISA measurements. The latter studies also showed a decrease in Rbp4 protein levels in the CSF of RRMS patients when compared with samples collected from individuals diagnosed with non-inflammatory diseases of the CNS. Since this protein binds TTR, it is tempting to speculate that the observed decrease in the abundance of Rbp4 in the CSF of patients with RRMS may be related to the observed oligomerization of TTR. Previous proteomics studies also reported a decrease in the abundance of Rbp4 in the CSF of patients with MS when compared with the clinically isolated syndrome [51], and with other neurological disorders (not validated) [17].

The proteomics studies also showed a downregulation in Alpha-1-antichymotrypsin in the CSF of RRMS patients when compared with samples collected from patients with other inflammatory diseases of the CNS. This is supported by the results obtained in validation studies using ELISA, in both genders. A previous study also reported a significant decrease in Alpha-1-antichymotrypsin protein levels in the CSF of patients with RRMS when compared with patients with other inflammatory neurological disorders [20]. Importantly, the alterations detected in the CSF were extended to the serum, particularly in males. This is an important observation in biomarker discovery, since blood can be obtained in a minimally invasive manner. Alpha-1-antichymotrypsin is a protease that is secreted from activated astrocytes [52, 53], as well as from the liver in response to acute inflammation [54]. Furthermore, Alpha-1-antichymotrypsin was detected in macrophages in MS lesions [55, 56].

Analysis of the CSF proteome also showed an upregulation of a spot identified as PGDS in samples from patients with RRMS, when individually compared with samples from the other two groups analyzed. This overall contrasts with the results obtained in the validation studies using ELISA, showing the opposite change in total protein abundance. Interestingly, this difference was also observed in samples from females but not from male patients. The differential results obtained in the two approaches suggest that PGDS may be post-translationally modified in the CSF of patients with RRMS, thus affecting the pattern of migration of the protein in 2D-PAGE, as it was detected in two different protein spots, and pointing to the presence of different proteoforms. PGDS is the most abundant brain-synthesized protein in the CSF and a previous proteomics study also showed an upregulation of this enzyme in CSF samples from RRMS patients when compared with patients showing clinically isolated syndrome, and other inflammatory disorders of the nervous system [57]. Furthermore, an upregulation in PGDS was detected in oligodendrocytes and hypertrophied astrocytes in the demyelinated plaques of patients with MS [58]. In contrast with the results obtained in the analysis of CSF samples, no differences were detected in PGDS protein levels in serum samples from the three groups of patients. Given the lower levels of PGDS in the serum when compared with the CSF, the ELISA method may not be sensitive enough to detect putative changes in the abundance of the protein.

The proteomic study also showed a downregulation of one protein spot identified as Gelsolin in CSF samples from patients with RRMS when compared with samples collected from individuals with other inflammatory diseases of the CNS. Similar results were observed in the ELISA assay, but only in females, while no differences were detected on males. A similar decrease in Gelsolin protein levels was detected in the CSF of patients with MS when compared to samples obtained from individuals with other neurological disorders [17, 59, 60]. Interestingly, in patients with MS, CSF Gelsolin undergoes glutathionylation [49], a posttranslational modification that alters protein function, interactions, and localization across physiological processes, and acts as a protective mechanism against oxidative damage [61]. Cytoplasmic Gelsolin drives the differentiation of oligodendrocyte precursor cells [62], suggesting that the downregulation of this actin-severing protein may play a role in the pathogenesis of MS. Although two previous studies reported a decrease in Gelsolin protein levels in the blood of patients with MS when compared with individuals with other disorders of the nervous system [60, 63], no differences were found in the present study.

Two protein spots identified as ApoE and Angiotensinogen were upregulated in the 2D-gels prepared with CSF samples from RRMS patients when compared with samples from individuals with other inflammatory diseases of the CNS. These results were not confirmed in the ELISA assays, suggesting that the proteins may also undergo posttranslational modifications in the CSF of RRMS patients without changing their total abundance. In accordance with the results obtained in the present work, a gel-based proteomics study also showed an upregulation in ApoE in the CSF of MS patients when compared with samples from patients with clinically isolated syndrome or with a group of individuals with no evidence of acute or chronic neurologic or systemic disease [64]. This contrasts with the results of two previous studies which reported an upregulation ApoE in the serum of MS patients during a relapse, when compared with the remission phase, while no differences were detected in the CSF [65, 66]. Therefore, at this point, a relation between ApoE changes in the CSF and serum and MS remains elusive. In accordance with the results obtained in the proteomics study herein reported, an upregulation in Angiotensinogen protein levels was also found in the CSF of patients with secondary progressive MS relative to patients with other neurological disorders [67].

A lower number of proteins was found to distinguish the CSF of patients with RRMS and individuals with non-inflammatory diseases of the nervous system when compared with the set of proteins that were differentially expressed between the former group and individuals with other inflammatory diseases of the nervous system. This is surprising given the larger differences in the etiology of the diseases in the former group. Gelsolin was one of the proteins identified in the 2D-gels that distinguished the CSF from RRMS patients from the other two groups of patients, being the spot volumetry downregulated under the former conditions. Although the results of the ELISA studies did not confirm the difference between the Gelsolin protein levels in the CSF of RRMS patients and in individuals with non-inflammatory diseases of the CNS, the data from the proteomics analysis suggest that a change in a posttranslational modification of the protein in the CSF, with impact on its migration in 2D-PAGE, is a hallmark of MS patients.

The results of the proteomics study also showed a significant decrease in Agrin protein levels in the CSF of RRMS patients when compared with the results obtained for individuals with non-inflammatory diseases of the CNS, which, however, could not be confirmed in the ELISA assay. Interestingly, serum Agrin protein levels were found to be significantly reduced in RRMS patients when compared with individuals with non-inflammatory diseases of the CNS, and similar results were obtained when samples from male patients were analyzed independently. Although the studies using 2D-PAGE did not show differences in Agrin content between the CSF of RRMS patients and individuals with other inflammatory diseases of the CNS, a significant decrease in the abundance of the protein in the former group was detected using the ELISA approach. The differences detected in the ELISA experiments may correspond to protein spots that were not detected in 2D-PAGE. There are numerous pieces of evidence showing roles for Agrin in synaptic development, plasticity and signaling in the brain [68], but less is known about its role in immune system regulation [69]. To the best of our knowledge, this is the first study reporting alterations in CSF and serum Agrin protein levels associated with MS. The mechanisms underlying these alterations remain to be identified.

2D-PAGE analysis of the CSF also identified two spots as EF-hand calcium-binding domain-containing protein 13 and ApoB which allowed distinguishing samples from RRMS patients, and the other two groups tested together. This is the first time that alterations in the abundance of EF-hand calcium-binding domain-containing protein 13 in the CSF are associated with RRMS and deserves further investigation in future work. Analysis of the serum ApoB protein levels in patients after the first demyelinating event also showed a direct correlation with the number of T2 lesions developed after 2 years, and its upregulation is correlated with increased disability in MS patients [70–72].

It is interesting to note a distinct pattern in the results obtained for the alterations in the CSF proteome in samples from RRMS patients when compared with the other two groups, as analyzed by ELISA and by 2D-PAGE. In the latter conditions some of the protein spots were downregulated in samples from RRMS patients, while for other proteins, it was observed an upregulation. In contrast, when ELISA was used to analyze the alterations in the CSF proteome in samples from patients with RRMS, there was a decrease or no effect in the total abundance of the proteins when compared with samples from the other two groups of patients. The differential results obtained with the two approaches are likely to result from specific post-translational modifications of the proteins which can be detected in specific protein spots in 2D-gels, without changing their total abundance as detected with ELISA.

Misdiagnosis occurs in roughly 50% of patients that have been initially diagnosed with MS [73]. For this reason, all patients included in the present study were retrospectively diagnosed with MS, according to the best clinical practices and diagnostic criteria. Moreover, the current revision of the McDonald criteria evolved in a way so that largely avoids diagnosing clinically healthy controls with MS. Most diseases identified by Salomon et al. [73] that mimicked MS were also identified in the overall population enrolled in the present study diagnosed with non-inflammatory and other inflammatory diseases of the CNS (e.g., Migraine, Fibromyalgia, NMO among others). Having all this together, we strongly believe that the MS patients tested in the present study have indeed been correctly diagnosed. In future work, it would be of most utility to test these biomarkers in a healthy population. Such study may also contribute to decrease the misdiagnosis of MS patients at an early stage of the disease.

## Conclusions

In conclusion, Alpha-1-antichymotrypsin, PGDS, TTR, ApoE and Gelsolin were identified as potential biomarkers for RRMS when compared with other inflammatory diseases of CNS. Agrin, Myosin-15 and Gelsolin were identified as putative biomarkers to distinguish RRMS from non-inflammatory diseases of the CNS. Furthermore, EF-hand calcium-binding domain-containing protein 13, Gelsolin and ApoB were identified as biomarkers to distinguish RRMS from inflammatory and non-inflammatory diseases of the CNS. This set of spots, when used in combination, allow a correct classification of 80–90% of the samples, showing the great potential of using a combination of protein markers in the diagnosis of RRMS. However, when validating the findings in the discovery group, only the levels of aggregated TTR showed potential to differentiate the RRMS patients from the other two groups included in this study. The machine learning techniques applied over the eight selected proteins showed the potential of TTR to discriminate patients with RRMS from the remaining patients. This points to the relevance of analyzing posttranslational modifications of the proteome in the identification of MS biomarkers in CSF-derived samples. Future studies focusing on the alterations in posttranslational modifications of the putative biomarkers identified in the present study are expected to contribute to the identification of a set of makers - proteins and their modifications- that may help in the characterization of MS patients and, most importantly, to help differentiating these patients from those with similar inflammatory diseases of the CNS.

## Supplementary Information

**Additional file 1: Figure S1.** Representative gel obtained using human CSF samples resolved by 2D-PAGE and stained with the Flamingo™ Fluorescence stain.

**Additional file 2: Figure S2.** Master gel images showing spots differentially expressed in the analyzed samples, as determined by the Student’s *t* test: (a) multiple sclerosis vs. other inflammatory diseases of the CNS; (b) multiple sclerosis vs. non-inflammatory diseases of the CNS; (c) multiple sclerosis vs. other inflammatory diseases and non-inflammatory diseases of CNS as a single group.

**Additional file 3: Figure S3.** Total abundance of ApoE in the CSF of patients diagnosed with Relapse–remitting multiple sclerosis (RRMS), non-inflammatory diseases of the CNS (N-INF), and with other inflammatory diseases of the CNS (INF), and further sub-divided by gender. The results represent the mean ± SEM and statistical analysis was performed by one-way ANOVA followed by the Tukey’s multiple comparison test, comparing all the indicated conditions. (ns, *p* > 0.05)

**Additional file 4: Figure S4.** Total abundance of Angiotensinogen in the CSF of patients diagnosed with Relapse–remitting multiple sclerosis (RRMS), non-inflammatory diseases of the CNS (N-INF), and with other inflammatory diseases of the CNS (INF), and further sub-divided by gender. The results represent the mean ± SEM and statistical analysis was performed by one-way ANOVA followed by the Tukey’s multiple comparison test, comparing all the indicated conditions. (ns, *p* > 0.05)

**Additional file 5: Figure S5.** Dendrogram representing the similarity between the protein content in the patients, regarding their clinical condition. RRMS stands for Relapse–remitting multiple sclerosis, INF for other inflammatory diseases of the CNS and N-INF corresponds to non-inflammatory diseases of the CNS.

**Additional file 6: Figure S6.** Biplots representing patients’ response to the proteins in study on (A) PC1 and PC2 recovering 46.5% of variance; (B) PC1 and PC3 recovering 41.3% and (C) PC2 and PC3 recovering approximately 32% of information variability.

**Additional file 7.** **Table S1-** Principal component analysis considering the results obtained in the proteomics analysis of samples collected from patients with Relapse-Remitting Multiple Sclerosis (RRMS) and other inflammatory diseases of the CNS (INF). **Table S2-** Principal component analysis considering the results obtained in the proteomics analysis of samples collected from patients with Relapse-Remitting Multiple Sclerosis (RRMS) and non-inflammatory diseases of the CNS (N-INF). **Table S3-** Protein spots showing a differential distribution between Relapse-Remitting Multiple Sclerosis (RRMS) and other inflammatory diseases of the CNS (INF). **Table S4-** Protein spots from CSF samples showing a differential distribution between Relapse-Remitting Multiple Sclerosis (RRMS) and non-inflammatory diseases of CNS (N-INF). **Table S5-** Protein spots showing a significant change in abundance when comparing CSF samples from relapse-remitting multiple sclerosis vs. other inflammatory and non-inflammatory diseases of the CNS (control) as a single group. **Table S6-** Protein Identification from selected 2D-PAGE spots (SSP) by LC-MS/MS. **Table S7-** Eigenvalues for the proteins in the study in the CSF dataset. The most relevant proteins are underlined and correspond to eigenvalues superior to 1. **Table S8-** Loadings of the PCA for CSF scaled data. Loadings with an absolute value ≥ 0.40 are underlined. **Table S9-** Means of the clusters present in the HCA dendrogram representing the similarity between the protein content in the CSF of the three groups of patients, as determined by ELISA.

## Data Availability

All data generated or analysed during this study are included in this published article [and its additional files]. Additional data sets analysed during the current study are available from the corresponding author on reasonable request.

## Abbreviations

ApoB: Apolipoprotein B-100
ApoE: Apolipoprotein E
CNS: Central nervous system
CSF: Cerebrospinal fluid
HCA: Hierarchical Cluster Analysis
INF: Inflammatory
LDA: Linear Discrimination Analysis
MS: Multiple sclerosis
N-INF: Non-inflammatory
PAGE: Polyacrylamide gel electrophoresis
PCA: Principal Component Analysis
PC: Principal component
PGDS: Prostaglandin-H2 D isomerase
RRMS: Relapse–remitting multiple sclerosis
Rbp4: Retinol-binding protein 4
TTR: Transthyretin
