# Supplementary figures and images for: Posttranslational modifications of proteins are key features in the identification of CSF biomarkers of multiple sclerosis
Source: J Neuroinflammation. 2022 Feb 8;19:44. doi: 10.1186/s12974-022-02404-2 (PMC8822857; doi:10.1186/s12974-022-02404-2)

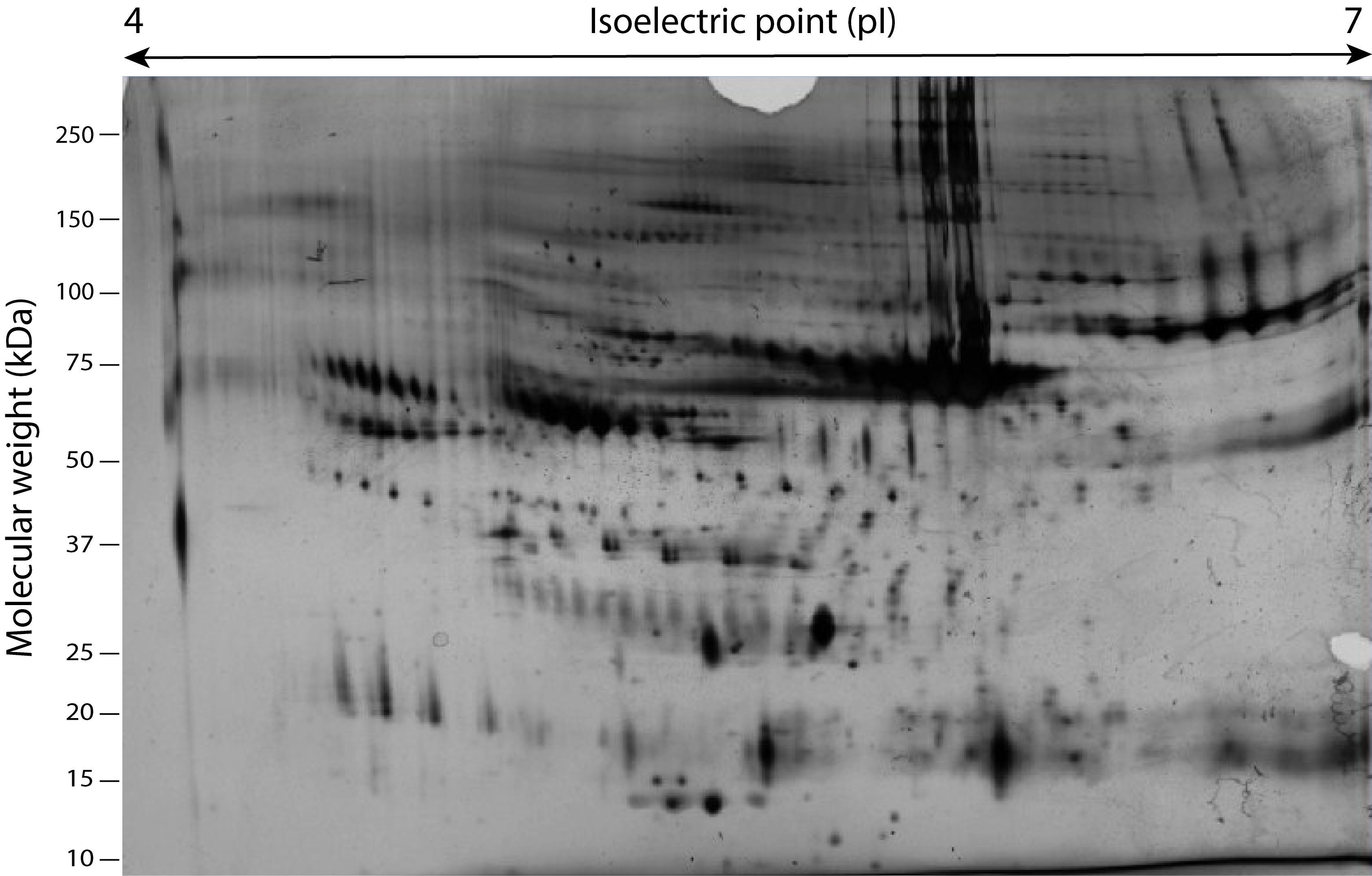

Supplement: Supplementary file 1 — Additional file 1: Figure S1. Representative gel obtained using human CSF samples resolved by 2D-PAGE and stained with the Flamingo™ Fluorescence stain. [file 12974_2022_2404_MOESM1_ESM.tif]

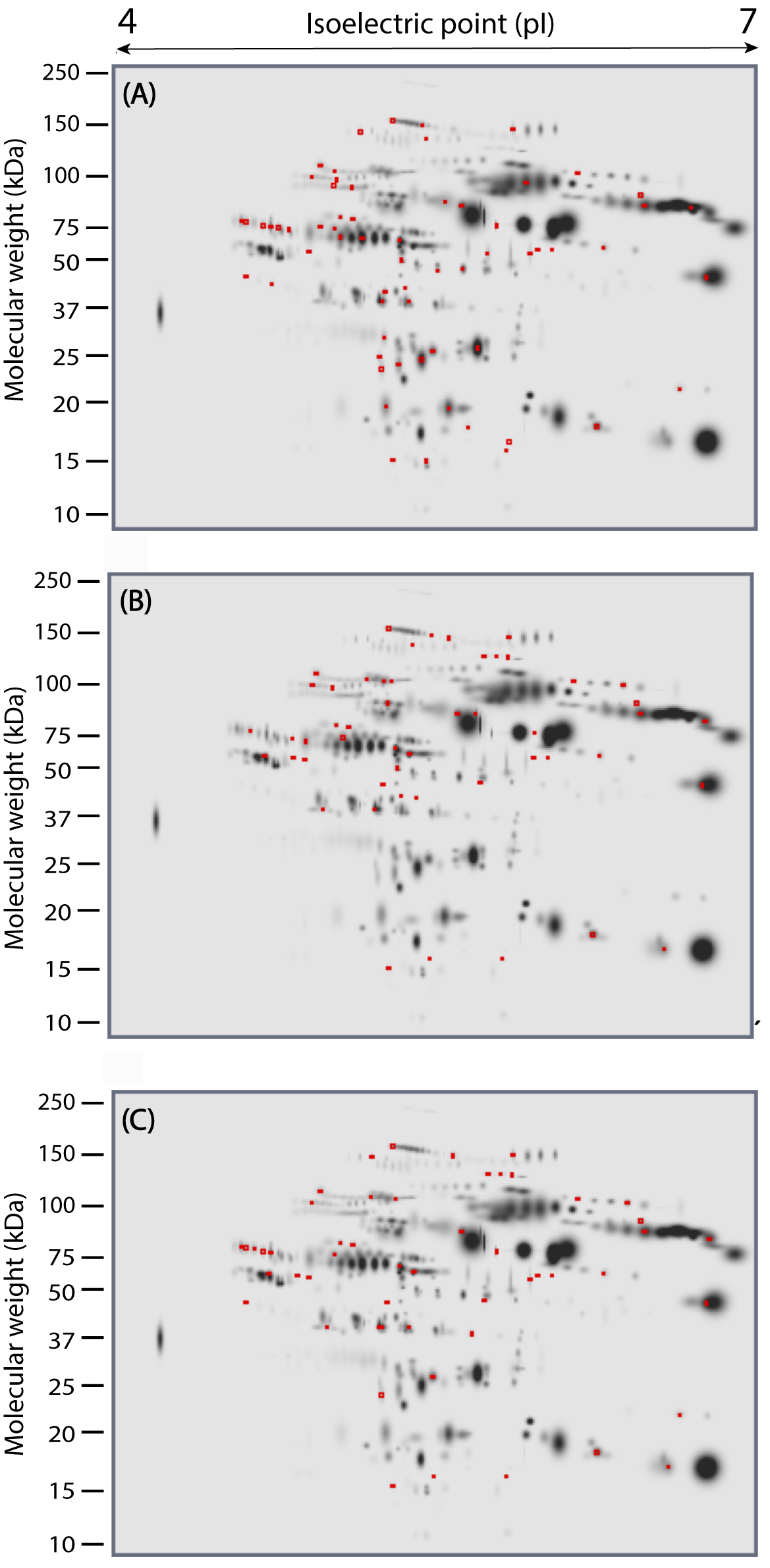

Supplement: Supplementary file 2 — Additional file 2: Figure S2. Master gel images showing spots differentially expressed in the analyzed samples, as determined by the Student’s t test: (a) multiple sclerosis vs. other inflammatory diseases of the CNS; (b) multiple sclerosis vs. non-inflammatory diseases of the CNS; (c) multiple sclerosis vs. other inflammatory diseases and non-inflammatory diseases of CNS as a single group. [file 12974_2022_2404_MOESM2_ESM.tif]

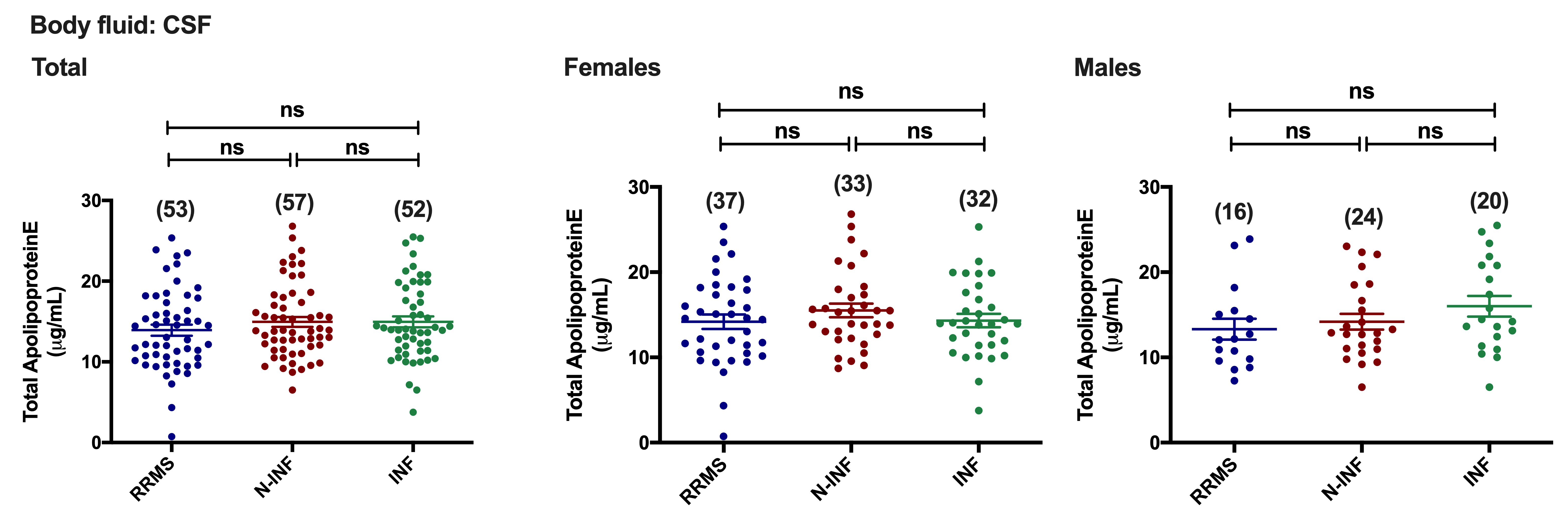

Supplement: Supplementary file 3 — Additional file 3: Figure S3. Total abundance of ApoE in the CSF of patients diagnosed with Relapse–remitting multiple sclerosis (RRMS), non-inflammatory diseases of the CNS (N-INF), and with other inflammatory diseases of the CNS (INF), and further sub-divided by gender. The results represent the mean ± SEM and statistical analysis was performed by one-way ANOVA followed by the Tukey’s multiple comparison test, comparing all the indicated conditions. (ns, p > 0.05) [file 12974_2022_2404_MOESM3_ESM.tiff]

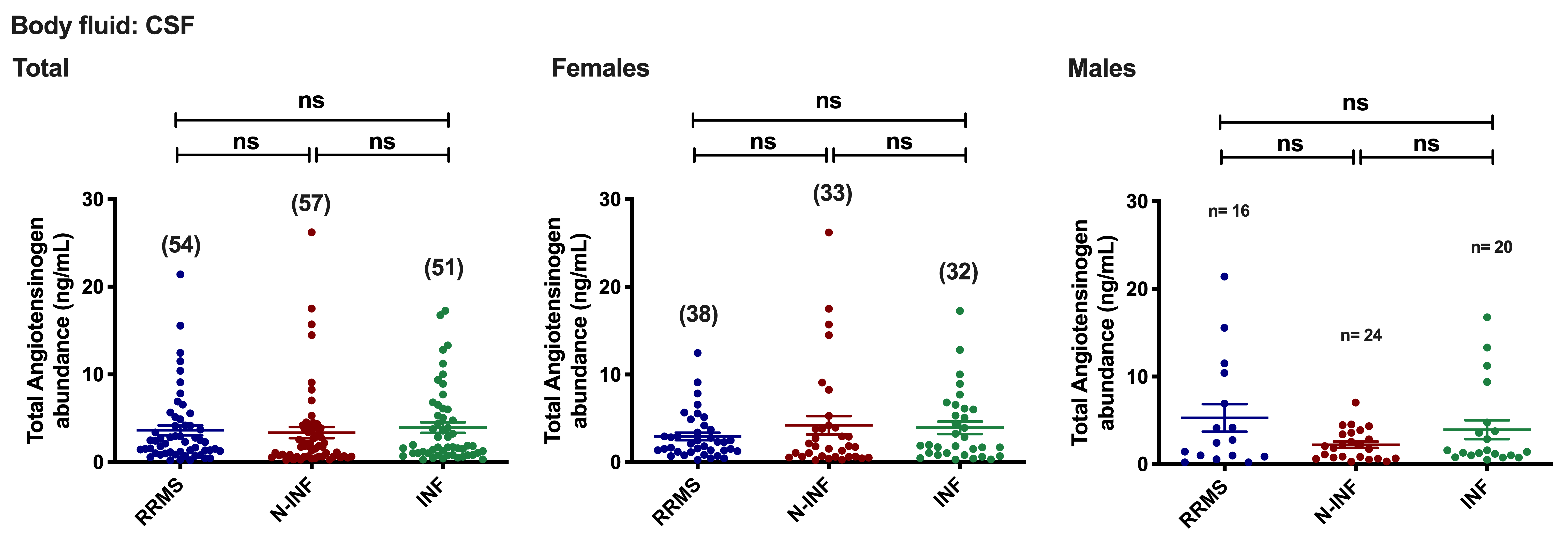

Supplement: Supplementary file 4 — Additional file 4: Figure S4. Total abundance of Angiotensinogen in the CSF of patients diagnosed with Relapse–remitting multiple sclerosis (RRMS), non-inflammatory diseases of the CNS (N-INF), and with other inflammatory diseases of the CNS (INF), and further sub-divided by gender. The results represent the mean ± SEM and statistical analysis was performed by one-way ANOVA followed by the Tukey’s multiple comparison test, comparing all the indicated conditions. (ns, p > 0.05) [file 12974_2022_2404_MOESM4_ESM.tiff]

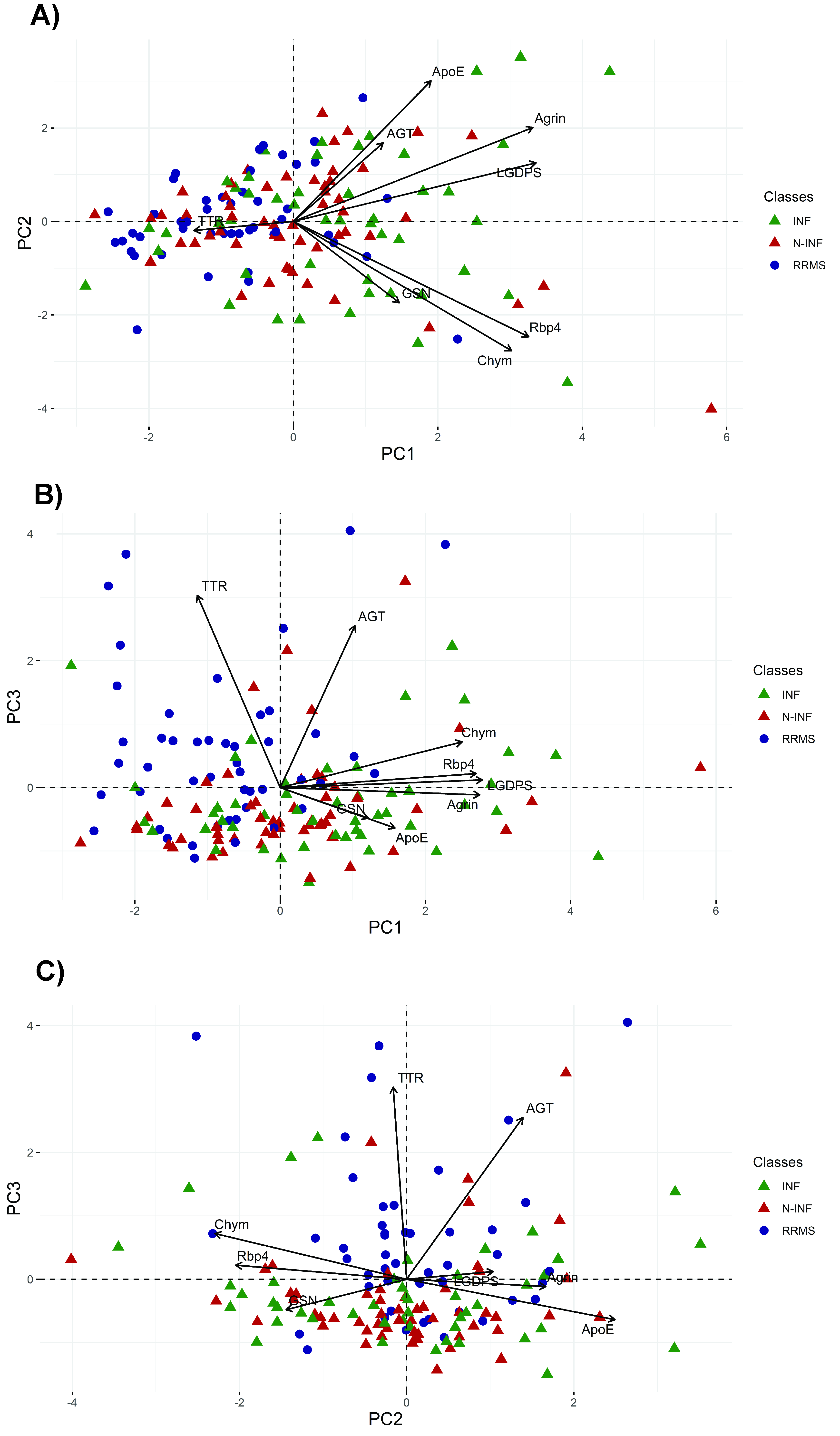

Supplement: Supplementary file 6 — Additional file 6: Figure S6. Biplots representing patients’ response to the proteins in study on (A) PC1 and PC2 recovering 46.5% of variance; (B) PC1 and PC3 recovering 41.3% and (C) PC2 and PC3 recovering approximately 32% of information variability. [file 12974_2022_2404_MOESM6_ESM.tif]
