# Supplementary material for: Posttranslational modifications of proteins are key features in the identification of CSF biomarkers of multiple sclerosis
Source: J Neuroinflammation. 2022 Feb 8;19:44. doi: 10.1186/s12974-022-02404-2 (PMC8822857; doi:10.1186/s12974-022-02404-2)
Supplement: Supplementary file 7 — Additional file 7. Table S1- Principal component analysis considering the results obtained in the proteomics analysis of samples collected from patients with Relapse-Remitting Multiple Sclerosis (RRMS) and other inflammatory diseases of the CNS (INF). Table S2- Principal component analysis considering the results obtained in the proteomics analysis of samples collected from patients with Relapse-Remitting Multiple Sclerosis (RRMS) and non-inflammatory diseases of the CNS (N-INF). Table S3- Protein spots showing a differential distribution between Relapse-Remitting Multiple Sclerosis (RRMS) and other inflammatory diseases of the CNS (INF). Table S4- Protein spots from CSF samples showing a differential distribution between Relapse-Remitting Multiple Sclerosis (RRMS) and non-inflammatory diseases of CNS (N-INF). Table S5- Protein spots showing a significant change in abundance when comparing CSF samples from relapse-remitting multiple sclerosis vs. other inflammatory and non-inflammatory diseases of the CNS (control) as a single group. Table S6- Protein Identification from selected 2D-PAGE spots (SSP) by LC-MS/MS. Table S7- Eigenvalues for the proteins in the study in the CSF dataset. The most relevant proteins are underlined and correspond to eigenvalues superior to 1. Table S8- Loadings of the PCA for CSF scaled data. Loadings with an absolute value ≥ 0.40 are underlined. Table S9- Means of the clusters present in the HCA dendrogram representing the similarity between the protein content in the CSF of the three groups of patients, as determined by ELISA. [file 12974_2022_2404_MOESM7_ESM.docx]

**Supplementary Material**

**Supplementary Table 1 -** Principal component analysis considering the results obtained in the proteomics analysis of samples collected from patients with Relapse-Remitting Multiple Sclerosis (RRMS) and other inflammatory diseases of the CNS (INF). For each component it is indicated the eigenvalues, % of variance and cumulative variance recovered (left). The same is presented for the eigenvalues in excess of 1 (right).

| Total Variance Explained | | | | | | |
| --- | --- | --- | --- | --- | --- | --- |
| Component | Initial Eigenvalues | | | Extraction Sums of Squared Loadings | | |
|  | Total | % of Variance | Cumulative % | Total | % of Variance | Cumulative % |
| 1 | 9.485 | 14.156 | 14.156 | 9.485 | 14.156 | 14.156 |
| 2 | 4.106 | 6.129 | 20.285 | 4.106 | 6.129 | 20.285 |
| 3 | 3.503 | 5.229 | 25.514 | 3.503 | 5.229 | 25.514 |
| 4 | 2.960 | 4.418 | 29.932 | 2.960 | 4.418 | 29.932 |
| 5 | 2.731 | 4.075 | 34.008 | 2.731 | 4.075 | 34.008 |
| 6 | 2.673 | 3.989 | 37.996 | 2.673 | 3.989 | 37.996 |
| 7 | 2.525 | 3.768 | 41.765 | 2.525 | 3.768 | 41.765 |
| 8 | 2.198 | 3.280 | 45.045 | 2.198 | 3.280 | 45.045 |
| 9 | 2.078 | 3.102 | 48.147 | 2.078 | 3.102 | 48.147 |
| 10 | 1.914 | 2.857 | 51.004 | 1.914 | 2.857 | 51.004 |
| 11 | 1.856 | 2.770 | 53.774 | 1.856 | 2.770 | 53.774 |
| 12 | 1.696 | 2.531 | 56.305 | 1.696 | 2.531 | 56.305 |
| 13 | 1.638 | 2.444 | 58.749 | 1.638 | 2.444 | 58.749 |
| 14 | 1.582 | 2.361 | 61.110 | 1.582 | 2.361 | 61.110 |
| 15 | 1.438 | 2.146 | 63.256 | 1.438 | 2.146 | 63.256 |
| 16 | 1.419 | 2.118 | 65.373 | 1.419 | 2.118 | 65.373 |
| 17 | 1.250 | 1.866 | 67.239 | 1.250 | 1.866 | 67.239 |
| 18 | 1.218 | 1.818 | 69.057 | 1.218 | 1.818 | 69.057 |
| 19 | 1.184 | 1.767 | 70.824 | 1.184 | 1.767 | 70.824 |
| 20 | 1.120 | 1.671 | 72.496 | 1.120 | 1.671 | 72.496 |
| 21 | 1.071 | 1.598 | 74.094 | 1.071 | 1.598 | 74.094 |
| 22 | 1.051 | 1.568 | 75.662 | 1.051 | 1.568 | 75.662 |
| 23 | .970 | 1.448 | 77.110 |  |  |  |
| 24 | .957 | 1.428 | 78.538 |  |  |  |
| 25 | .902 | 1.346 | 79.884 |  |  |  |
| 26 | .879 | 1.311 | 81.195 |  |  |  |
| 27 | .819 | 1.222 | 82.417 |  |  |  |
| 28 | .799 | 1.193 | 83.610 |  |  |  |
| 29 | .751 | 1.120 | 84.730 |  |  |  |
| 30 | .712 | 1.063 | 85.794 |  |  |  |
| 31 | .678 | 1.011 | 86.805 |  |  |  |
| 32 | .602 | .898 | 87.703 |  |  |  |
| 33 | .581 | .867 | 88.570 |  |  |  |
| 34 | .551 | .822 | 89.392 |  |  |  |
| 35 | .529 | .790 | 90.182 |  |  |  |
| 36 | .504 | .752 | 90.934 |  |  |  |
| 37 | .461 | .688 | 91.621 |  |  |  |
| 38 | .443 | .662 | 92.283 |  |  |  |
| 39 | .410 | .611 | 92.894 |  |  |  |
| 40 | .408 | .609 | 93.504 |  |  |  |
| 41 | .390 | .582 | 94.085 |  |  |  |
| 42 | .361 | .540 | 94.625 |  |  |  |
| 43 | .345 | .515 | 95.140 |  |  |  |
| 44 | .334 | .498 | 95.638 |  |  |  |
| 45 | .273 | .407 | 96.045 |  |  |  |
| 46 | .257 | .383 | 96.428 |  |  |  |
| 47 | .239 | .357 | 96.785 |  |  |  |
| 48 | .231 | .344 | 97.129 |  |  |  |
| 49 | .214 | .320 | 97.449 |  |  |  |
| 50 | .195 | .291 | 97.740 |  |  |  |
| 51 | .178 | .266 | 98.006 |  |  |  |
| 52 | .167 | .249 | 98.254 |  |  |  |
| 53 | .153 | .228 | 98.482 |  |  |  |
| 54 | .146 | .218 | 98.700 |  |  |  |
| 55 | .129 | .193 | 98.893 |  |  |  |
| 56 | .116 | .173 | 99.066 |  |  |  |
| 57 | .102 | .152 | 99.217 |  |  |  |
| 58 | .096 | .144 | 99.361 |  |  |  |
| 59 | .075 | .112 | 99.473 |  |  |  |
| 60 | .072 | .108 | 99.581 |  |  |  |
| 61 | .068 | .101 | 99.681 |  |  |  |
| 62 | .049 | .073 | 99.755 |  |  |  |
| 63 | .044 | .066 | 99.820 |  |  |  |
| 64 | .041 | .061 | 99.881 |  |  |  |
| 65 | .032 | .047 | 99.929 |  |  |  |
| 66 | .026 | .039 | 99.968 |  |  |  |
| 67 | .022 | .032 | 100.000 |  |  |  |
| Extraction Method: Principal Component Analysis. | | | | | | |

**Supplementary Table 2 -** Principal component analysis considering the results obtained in the proteomics analysis of samples collected from patients with Relapse-Remitting Multiple Sclerosis (RRMS) and non-inflammatory diseases of the CNS (N-INF). For each component it is indicated the eigenvalues, % of variance and cumulative variance recovered (left). The same is presented for the eigenvalues in excess of 1 (right).

| **Total Variance Explained** | | | | | | |
| --- | --- | --- | --- | --- | --- | --- |
| Component | Initial Eigenvalues | | | Extraction Sums of Squared Loadings | | |
|  | Total | % of Variance | Cumulative % | Total | % of Variance | Cumulative % |
| 1 | 5.660 | 11.320 | 11.320 | 5.660 | 11.320 | 11.320 |
| 2 | 3.534 | 7.068 | 18.387 | 3.534 | 7.068 | 18.387 |
| 3 | 2.441 | 4.881 | 23.269 | 2.441 | 4.881 | 23.269 |
| 4 | 2.413 | 4.827 | 28.095 | 2.413 | 4.827 | 28.095 |
| 5 | 2.170 | 4.339 | 32.435 | 2.170 | 4.339 | 32.435 |
| 6 | 1.970 | 3.940 | 36.374 | 1.970 | 3.940 | 36.374 |
| 7 | 1.782 | 3.565 | 39.939 | 1.782 | 3.565 | 39.939 |
| 8 | 1.722 | 3.444 | 43.383 | 1.722 | 3.444 | 43.383 |
| 9 | 1.626 | 3.253 | 46.636 | 1.626 | 3.253 | 46.636 |
| 10 | 1.569 | 3.138 | 49.774 | 1.569 | 3.138 | 49.774 |
| 11 | 1.517 | 3.035 | 52.809 | 1.517 | 3.035 | 52.809 |
| 12 | 1.473 | 2.945 | 55.754 | 1.473 | 2.945 | 55.754 |
| 13 | 1.403 | 2.806 | 58.560 | 1.403 | 2.806 | 58.560 |
| 14 | 1.247 | 2.495 | 61.055 | 1.247 | 2.495 | 61.055 |
| 15 | 1.212 | 2.425 | 63.480 | 1.212 | 2.425 | 63.480 |
| 16 | 1.185 | 2.369 | 65.849 | 1.185 | 2.369 | 65.849 |
| 17 | 1.129 | 2.258 | 68.107 | 1.129 | 2.258 | 68.107 |
| 18 | 1.050 | 2.101 | 70.208 | 1.050 | 2.101 | 70.208 |
| 19 | 1.012 | 2.025 | 72.232 | 1.012 | 2.025 | 72.232 |
| 20 | .959 | 1.918 | 74.150 |  |  |  |
| 21 | .929 | 1.857 | 76.008 |  |  |  |
| 22 | .842 | 1.683 | 77.691 |  |  |  |
| 23 | .819 | 1.639 | 79.329 |  |  |  |
| 24 | .738 | 1.475 | 80.804 |  |  |  |
| 25 | .715 | 1.430 | 82.234 |  |  |  |
| 26 | .696 | 1.392 | 83.626 |  |  |  |
| 27 | .663 | 1.326 | 84.952 |  |  |  |
| 28 | .622 | 1.245 | 86.197 |  |  |  |
| 29 | .593 | 1.187 | 87.383 |  |  |  |
| 30 | .590 | 1.180 | 88.563 |  |  |  |
| 31 | .510 | 1.020 | 89.584 |  |  |  |
| 32 | .474 | .948 | 90.532 |  |  |  |
| 33 | .458 | .917 | 91.449 |  |  |  |
| 34 | .442 | .884 | 92.333 |  |  |  |
| 35 | .431 | .862 | 93.195 |  |  |  |
| 36 | .381 | .762 | 93.957 |  |  |  |
| 37 | .375 | .751 | 94.707 |  |  |  |
| 38 | .341 | .682 | 95.389 |  |  |  |
| 39 | .317 | .634 | 96.022 |  |  |  |
| 40 | .299 | .597 | 96.620 |  |  |  |
| 41 | .275 | .551 | 97.170 |  |  |  |
| 42 | .262 | .525 | 97.695 |  |  |  |
| 43 | .215 | .429 | 98.125 |  |  |  |
| 44 | .180 | .361 | 98.485 |  |  |  |
| 45 | .170 | .340 | 98.825 |  |  |  |
| 46 | .161 | .322 | 99.148 |  |  |  |
| 47 | .149 | .297 | 99.445 |  |  |  |
| 48 | .120 | .241 | 99.685 |  |  |  |
| 49 | .092 | .184 | 99.869 |  |  |  |
| 50 | .066 | .131 | 100.000 |  |  |  |
| Extraction Method: Principal Component Analysis. | | | | | | |

**Supplementary Table 3** – Protein spots showing a differential distribution between Relapse-Remitting Multiple Sclerosis (RRMS) and other inflammatory diseases of the CNS (INF).

| **SSP** | | **RRMS**  **(relative volume)** | **INF**  **(relative volume)** | **p-value** | **Fold change** | **Mass**  **(Daltons)** | **Identity** |
| --- | --- | --- | --- | --- | --- | --- | --- |
| 1502 | | 523,307.4↓ | 739,414.0 | 0.004 | 0.708 | 47,792 | Alpha-1-antichymotrypsin |
| 1703 | | 177,513.4↑ | 37,243.5 | 1.233E-23 | 4.766 | - | - |
| 3004 | | 2,036,025.0↑ | 1,5766,00.0 | 0.030 | 1.291 | 21,243 | Prostaglandin-H2  D-isomerase |
| 3006 | | 55,053.4↓ | 262,900.8 | 0.001 | 0.209 | 23,451 | Retinol binding protein 4 |
| 3103 | | 4,958,438.0↑ | 4,024,761.0 | 0.010 | 1.232 | 15,991 | Transthyretin |
| 4101 | | 980,143.2↑ | 654,237.1 | 4.254E-4 | 1.498 | 36,246 | Apolipoprotein E |
| 7404 |  | 277,019.2↑ | 74,473.28 | 1.467E-13 | 3.720 | 71,981 | Serum albumin |
| 7811 | | 220,366.2↓ | 320,338.8 | 2.012E-7 | 0.688 | 86,176 | Gelsolin |
| 8301 | | 486,403.2↑ | 247,322.3 | 3.383E-4 | 1.967 | 53,501 | Angiotensinogen |

RRMS- Relapse-Remitting Multiple Sclerosis; INF- other inflammatory diseases of CNS

**Supplementary Table 4** – Protein spots from CSF samples showing a differential distribution between Relapse-Remitting Multiple Sclerosis (RRMS) and non-inflammatory diseases of CNS (N-INF).

| **SSP** | **RRMS**  **(relative volume)** | **N-INF**  **(relative volume)** | **p-value** | **Fold change** | **Mass**  **(Daltons)** | **Identity** |
| --- | --- | --- | --- | --- | --- | --- |
| 5005 | 23,955.4↓ | 40,555.0 | 7.491E-7 | 0.591 | 22,557 | Agrin |
| 7404 | 277,019.2↑ | 141,943.5 | 9.676E-5 | 1.952 | 71,981 | Serum albumin |
| 7807 | 170,555.9↑ | 126,575.1 | 2.936E-5 | 1.347 | 225,930 | Myosin-15 |
| 7811 | 220,366.2↓ | 378,572.5 | 3.513E-5 | 0.582 | 86,176 | Gelsolin |
| 8006 | 2,278,850.0↑ | 1,358,579.0 | 3.031E-8 | 1.680 | 21,243 | Prostagladin-H2-D-isomerase |

RRMS- Relapse-Remitting Multiple Sclerosis; N-INF- non-inflammatory diseases of CNS

**Supplementary Table 5** – Protein spots showing a significant change in abundance when comparing CSF samples from relapse-remitting multiple sclerosis vs. other inflammatory and non-inflammatory diseases of the CNS (control) as a single group.

| **SSP** | **RRMS**  **(relative volume)** | **Control**  **(relative volume)** | **p-value** | **Fold change** | **Mass**  **(Daltons)** | **Identity** |
| --- | --- | --- | --- | --- | --- | --- |
| 7001 | 1,327,640.0↑ | 743,105.0 | 1.002E-6 | 1.787 | 111,143 | EF-hand calcium-binding domain- containing protein 13 |
| 7404 | 277,019.2↑ | 115,542.1 | 1.111E-8 | 2.398 | 71,981 | Serum albumin |
| 7811 | 220,366.2↓ | 355,785.4 | 9.943E-8 | 0.619 | 86,176 | Gelsolin |
| 8402 | 276,075.6↑ | 116,137.1 | 2.208E-3 | 2.377 | 51,714 | Apolipoprotein B-100 |

RRMS- Relapse-Remitting Multiple Sclerosis; Control- Other inflammatory and Non-inflammatory diseases of the CNS as a unique group

**Supplementary Table 6** - Protein Identification from selected 2D-PAGE spots (SSP) by LC-MS/MS.

| **SPP** | **Accession Number** | **Protein Name** | **Peptide Confidence** | **Peptide sequence** |
| --- | --- | --- | --- | --- |
| 1502 | P01011 | Alpha-1-antichymotrypsin | 99 | ADLSGITGAR |
|  |  |  | 99 | AVLDVFEEGTEASAATAVK |
|  |  |  | 99 | DEELSCTVVELK |
|  |  |  | 99 | EIGELYLPK |
|  |  |  | 99 | ITLLSALVETR |
|  |  |  | 99 | LYGSEAFATDFQDSAAAK |
|  |  |  | 99 | MEEVEAMLLPETLK |
|  |  |  | 99 | MEEVEAMLLPETLKR |
|  |  |  | 99 | NLAVSQVVHK |
|  |  |  | 99 | RLYGSEAFATDFQDSAAAK |
|  |  |  | 99 | WEMPFDPQDTHQSR |
|  |  |  | 99 | HPNSPLDEENLTQENQDR |
|  |  |  | 99 | KLINDYVK |
|  |  |  | 97 | LINDYVK |
| 3004 | P41222 | Prostaglandin-H2  D-isomerase | 99 | TMLLQPAGSLGSYSYR |
|  |  |  | 99 | AQGFTEDTIVFLPQTDK |
|  |  |  | 99 | APEAQVSVQPNFQQDK |
| 3006 | P02753 | Retinol-binding  protein 4 | 40* | YWGVASFLQK |
| 3103 | P02766 | Transthyretin | 99 | AADDTWEPFASGK |
|  |  |  | 99 | ALGISPFHEHAEVVFTANDSGPR |
|  |  |  | 99 | GSPAINVAVHVFR |
|  |  |  | 99 | KAADDTWEPFASGK |
|  |  |  | 99 | YTIAALLSPYSYSTTAVVTNPK |
|  |  |  | 99 | TSESGELHGLTTEEEFVEGIYK |
| 4101 | P02649 | Apolipoprotein E | 99 | AATVGSLAGQPLQER |
|  |  |  | 99 | AKLEEQAQQIR |
|  |  |  | 99 | AYKSELEEQLTPVAEETR |
|  |  |  | 99 | DRLDEVKEQVAEVR |
|  |  |  | 99 | GEVQAMLGQSTEELR |
|  |  |  | 99 | KVEQAVETEPEPELR |
|  |  |  | 99 | LAVYQAGAR |
|  |  |  | 99 | LEEQAQQIR |
|  |  |  | 99 | LGPLVEQGR |
|  |  |  | 99 | LKSWFEPLVEDMQR |
|  |  |  | 99 | LQAEAFQAR |
|  |  |  | 99 | LVQYRGEVQAMLGQSTEELR |
|  |  |  | 99 | SELEEQLTPVAEETR |
|  |  |  | 99 | VQAAVGTSAAPVPSDNH |
|  |  |  | 99 | SWFEPLVEDMQR |
|  |  |  |  |  |
|  |  |  |  |  |
| 5005 | O00468 | Agrin | 99 | AYGTGFVGCLR |
|  |  |  | 99 | SAGDVDTLAFDGR |
|  |  |  | 99 | ALQSNHFELSLR |
| 7001 | Q8IY85 | EF-hand calcium-binding domain-containing protein 13 | 32* | IMENDDLESKRPK |
|  |  |  |  |  |
| 7404 | P02768 | Albumin | 78* | AVMDDFAAFVEK |
|  |  |  | 32* | VFDEFKPLVEEPQNLIK |
|  |  | Ig gamma-1 chain C region | 33* | VVSVLTVLHQDWLNGK |
| 7807 | Q9Y2K3 | Myosin-15 | 42* | MKYENNVIQR |
| 7811 | P06396 | Gelsolin | 99 | AGALNSNDAFVLK |
|  |  |  | 99 | AQPVQVAEGSEPDGFWEALGGK |
|  |  |  | 99 | EPGLQIWR |
|  |  |  | 99 | VHVSEEGTEPEAMLQVLGPK |
|  |  |  | 99 | EVQGFESATFLGYFK |
|  |  |  | 99 | DPDQTDGLGLSYLSSHIANVER |
|  |  |  | 99 | PNSMVVEHPEFLK |
|  | P02768 | Albumin | 99 | AVMDDFAAFVEK |
|  |  |  | 99 | FQNALLVR |
|  |  |  | 99 | KVPQVSTPTLVEVSR |
|  |  |  | 99 | LVNEVTEFAK |
|  |  |  | 99 | SHCIAEVENDEMPADLPSLAADFVESK |
| 8006 | P41222 | Prostaglandin-H2  D-isomerase | 99 | APEAQVSVQPNFQQDK |
|  |  |  | 99 | TMLLQPAGSLGSYSYR |
|  |  |  | 99 | SPHWGSTYSVSVVETDYDQYALLYSQGSK |
| 8301 | P01019 | Angiotensinogen | 99 | DPTFIPAPIQAK |
|  |  |  | 99 | FMQAVTGWK |
|  |  |  | 99 | LQAILGVPWK |
|  |  |  | 99 | PKDPTFIPAPIQAK |
|  |  |  | 99 | ALQDQLVLVAAK |
| 8402 | P04114 | Apolipoprotein B-100 | 35* | VIGNMGQTMEQLTPELK |

Protein identification was performed using Protein Pilot v5 with peptide confidence above 95%, or using Mascot. *Mascot peptide score indicate identity or extensive homology (p<0.05).

**Supplementary Table 7** – Eigenvalues for the proteins in the study in the CSF dataset. The most relevant proteins are underlined and correspond to eigenvalues superior to 1.

|  | **Eigenvalue** | **% of variance** | **cumulative**  **% of variance** |
| --- | --- | --- | --- |
| **comp 1** | **2.229** | **27.857** | **27.857** |
| **comp 2** | **1.490** | **18.631** | **46.478** |
| **comp 3** | **1.072** | **13.396** | **59.883** |
| **comp 4** | 0.957 | 11.964 | 71.848 |
| **comp 5** | 0.820 | 10.246 | 82.094 |
| **comp 6** | 0.634 | 7.927 | 90.021 |
| **comp 7** | 0.449 | 5.609 | 95.630 |
| **comp 8** | 0.350 | 4.370 | 100.000 |

**Supplementary Table 8** – Loadings of the PCA for CSF scaled data. Loadings with an absolute value ≥ 0.40 are underlined.

|  | **PC1** | **PC2** | **PC3** | **PC4** | **PC5** | **PC6** | **PC7** | **PC8** |
| --- | --- | --- | --- | --- | --- | --- | --- | --- |
| **Chym** | **0.422** | **-0.473** | 0.175 | -0.210 | 0.001 | 0.161 | -0.212 | 0.673 |
| **LGDPS** | **0.469** | 0.214 | 0.029 | 0.275 | -0.401 | -0.299 | 0.607 | 0.197 |
| **Rbp4** | **0.455** | **-0.422** | 0.054 | -0.211 | 0.014 | 0.285 | 0.251 | -0.650 |
| **TTR** | -0.192 | -0.033 | **0.736** | 0.370 | -0.402 | 0.313 | -0.124 | -0.092 |
| **Agrin** | **0.463** | 0.343 | -0.028 | -0.105 | -0.313 | -0.226 | -0.673 | -0.232 |
| **ApoE** | 0.266 | **0.514** | -0.155 | 0.174 | 0.242 | 0.733 | 0.024 | 0.120 |
| **GSN** | 0.205 | -0.297 | -0.117 | 0.789 | 0.382 | -0.183 | -0.216 | -0.085 |
| **AGT** | 0.174 | 0.28672890 | **0.620** | -0.187 | 0.612 | -0.289 | 0.092 | -0.030 |

Chym, Alpha-1-antichymotrypsin; PGDS, Prostaglandin-D-isomerase; Rbp4, Retinol binding protein 4; TTR, Transthyretin; ApoE, Apolipoprotein E; GSN, Gelsolin; AGT, Angiotensinogen.

**Supplementary Table 9** – Means of the clusters present in the HCA dendrogram representing the similarity between the protein content in the CSF of the three groups of patients, as determined by ELISA.

| Group.1 | CHYM | LGDPS | Rbp4 | TTR | Agrin | ApoE | GSN | AGT | Figure 12 |
| --- | --- | --- | --- | --- | --- | --- | --- | --- | --- |
| 1 | -0.172 | -0.508 | -0.239 | -0.375 | -0.454 | -0.723 | -0.478 | -0.423 | B2 |
| 2 | -0.394 | -0.217 | -0.565 | 1.481 | -0.552 | -0.021 | -0.054 | -0.226 | B1 |
| 3 | 0.153 | 0.075 | 0.371 | -0.363 | 0.273 | 0.193 | 1.845 | -0.141 | B4 |
| 4 | -0.030 | 0.297 | 0.094 | -0.307 | 0.336 | 0.575 | -0.233 | 0.353 | B6 |
| 5 | 3.376 | 0.497 | 2.982 | 0.154 | 0.036 | -0.423 | 0.381 | 1.222 | B3 |
| 6 | -0.002 | 2.644 | -0.112 | -0.573 | 3.464 | 0.995 | -0.707 | 1.195 | B5 |
